# Multi-Source Co-adaptation for EEG-Based Emotion Recognition by Mining Correlation Information

**DOI:** 10.3389/fnins.2021.677106

**Published:** 2021-05-13

**Authors:** Jianwen Tao, Yufang Dan

**Affiliations:** Institute of Artificial Intelligence Application, Ningbo Polytechnic, Zhejiang, China

**Keywords:** electroencephalogram, emotion recognition, multi-source adaptation, feature selection, maximum mean discrepancy

## Abstract

Since each individual subject may present completely different encephalogram (EEG) patterns with respect to other subjects, existing subject-independent emotion classifiers trained on data sampled from cross-subjects or cross-dataset generally fail to achieve sound accuracy. In this scenario, the domain adaptation technique could be employed to address this problem, which has recently got extensive attention due to its effectiveness on cross-distribution learning. Focusing on cross-subject or cross-dataset automated emotion recognition with EEG features, we propose in this article a robust multi-source co-adaptation framework by mining diverse correlation information (MACI) among domains and features with *l*_2,1_−*norm* as well as correlation metric regularization. Specifically, by minimizing the statistical and semantic distribution differences between source and target domains, multiple subject-invariant classifiers can be learned together in a joint framework, which can make MACI use relevant knowledge from multiple sources by exploiting the developed correlation metric function. Comprehensive experimental evidence on DEAP and SEED datasets verifies the better performance of MACI in EEG-based emotion recognition.

## Introduction

Although emotion can be easily captured by human beings due to its close relationship with human’s cognition (Dolan, 2002), it cannot be readily recognized by instruments due to its complexity. Recently, as one of the most active research topics from the affective computing community, affection recognition had obtained a large amount of attention from pattern recognition and machine vision research fields (Kim et al., 2013). Generally, there are two categories on the responses of human emotion, i.e., external and internal responses. In this work, we focus on the latter. Basically, the representative internal responses include blood pressure, heart rate, respiration rate, electroencephalography (EEG), magneto encephalogram (Mühl et al., 2014), etc. Usually, the core components of a traditional emotion recognition system based on EEG are feature extraction and emotion classification (Lan et al., 2018). Practically, the time domain, frequency domain, and time–frequency domain are the main sources of the extracted EEG features (Jenke et al., 2014; Zhang et al., 2020b). The EEG feature extraction methods are more comprehensively reviewed in Jenke et al. (2014). In the past few years, aiming at the problem of emotion classification, a large number of emotion recognition models based on EEG signals have been proposed (Musha et al., 1997; Kim et al., 2013). For instance, a new group sparse canonical correlation analysis method was proposed in Zheng (2017) for simultaneous EEG channel selection and emotion recognition; in Li et al. (2018c), a graph regularized sparse linear regression method was proposed to deal with EEG-based emotion recognition. In the recent years, the deep learning method based on EEG has shown better performance than those traditional methods in emotion recognition and widely exploited in feature extraction and emotion recognition at the same time. For example, Zheng et al. (2015) employed deep belief network for EEG-based emotion recognition; Song et al. (2018) modeled the multi-channel EEG features by utilizing a graph and then performed EEG-based emotion recognition on these features; the work by Li et al. (2018b) had proposed a novel neural network model for EEG-based emotion recognition task.

While many models and methods for emotion recognition based on EEG have been proposed, most of them worked well only in the scenario that the training and test data were from the same distribution or domain. Under this hypothesis, the classifier trained on the source domain can directly predict the labels of the target data. However, for the problem of cross-domain emotion recognition based on EEG, many EEG-based emotion recognition methods would fail because of the distribution mismatch of EEG features. To this end, domain adaptation (DA) emotion recognition algorithms have emerged to investigate and address the automated emotion recognition problem (Chu et al., 2017), in which one has target domain with few or even none of labeled images by leveraging other related but different source/auxiliary domain(s) (Bruzzone and Marconcini, 2010). A typical example is the cross-subject EEG emotion recognition problem, in which the training and testing EEG data are from different subjects. To deal with the challenging cross-subject EEG emotion recognition problem, Pandey and Seeja (2019) proposed a subject-independent approach for EEG emotion recognition. Li et al. (2018a) proposed another method for cross-subject EEG emotion recognition. In the past decade, deep neural networks (Ganin et al., 2016; Li et al., 2018b) have also driven rapid progress in DA. The DA issues can be solved by the domain adversarial neural network (Ganin et al., 2016). It remains unclear, however, whether the performance of deep DA methods is really contributed by their deep feature representation, the fine-tuned classifiers, or is rather an outcome of the adaptation regularization terms (Ghifary et al., 2017).

Although the existing DA method has obvious effectiveness and efficiency in the special use of emotion recognition (Chu et al., 2017) in order to make use of the correlation knowledge among domains and features, there is little work to use the joint feature selection method and then carry out the multi-source adaptive domain recognition of cross-dataset. Besides this, during DA, most of the multi-source domain adaptation (MDA) methods (Yang et al., 2007; Tommasi et al., 2014) generally cope with the sources independently without considering the correlation information among the source domains, which may destroy the discriminant structure (either intrinsic or extrinsic) of multi-source domains. Last but not the least, for a MDA system, it is crucial for source weight determination during learning based on the correlation and quality of source domains. To the best of our knowledge, these characters are not feasible enough in extant MDA methods.

In order to solve the above-mentioned problems in existing MDA, we exploit the relevant information among sources and features to learn a multi-source emotion recognition model. We mainly adopt the strategy of digging the relationship between multi-source domains and one target domain (including feature and distribution) for promoting multi-source adaptive emotion recognition. We aim to progress beyond existing works that have partially addressed those issues by exploring to solve all the above-mentioned issues in a unified framework. Specifically, we develop a robust multi-source co-adaptation method for EEG-based emotion recognition by employing the correlation information (MACI) among features and sources *via l*_2,1_−*norm* (Nie et al., 2010a) and correlation metric regularization. Under this framework, the correlation metric function is developed to mine the invariant knowledge among multi-source domains, the *l*_2,1_−*norm* loss function aims to reduce the influence of outliers or noise, and row sparsity is designed to obtain the solution of sparse feature selection (Zhang et al., 2020b). We match distributions between each domain pair (including both target and multi-source domains) by minimizing the nonparametric maximum mean discrepancy (MMD) (Gretton et al., 2009; Pan et al., 2011) in a reproducing kernel Hilbert space (RKHS). The contributions of this article are listed as follows:

1. We propose a unified multi-source adaptive emotion recognition framework with EEG features by combining *l*_2,1_−*norm* and correlation metric regularization.
2. Our framework selects features in a collaborative way and considers the correlated knowledge among features; the importance of each feature does not need to be evaluated separately. In addition, in our unified framework, we can learn multiple functions of feature selection for all source adaptation subjects synchronously so that our framework can use the correlated information of multiple sources as auxiliary information.
3. In this framework, the original geometric structure is retained by using the graph Laplacian regularization, and the *l*_2,1_−*norm* minimization sparse regression approach is used to suppress the influence of noise or outliers in the domains, which shows the robustness of the framework.
4. Through a large number of experiments on two EEG datasets, we prove the effectiveness and convergence of this framework.

The remainder of the paper is organized as follows. In section “Related Work,” we discussed the related works with feature selection and MDA learning. In section “Proposed Framework,” our framework MACI will be designed, while section “Algorithm” arranges the corresponding optimal algorithm of MACI. Section “Algorithm Analysis” gives algorithm analysis, including the convergence and generalization. The experimental results and analysis on two real EEG datasets are presented in section “Experiments.” Finally, we conclude in section “Conclusion.”

## Related Work

In this section, we briefly review the prior emotion recognition with respect to EEG and multi-source adaptation techniques due to their relationships with our main ideas.

### Multi-Source Domain Adaptation

In the past several years, the mismatch problem between source and target domains has been solved by many DA technologies, which are widely used in a large number of visual applications, such as image annotation/classification, video concept detection, target recognition, and so on (Yang et al., 2007; Duan et al., 2012b, c; Tao et al., 2015, 2016, 2017; Ghifary et al., 2017). In the existing works for conquering DA, discovering one or multiple domain-invariant classifier(s) is a widely research topic (Yang et al., 2007; Tommasi et al., 2014) by constructing certain common subspace to make different sources have the same (or similar) marginal distributions. Therefore, the source classifier would obtain well performance on the target domain. Several methods have been studied to measure the distribution similarities (Gretton et al., 2009), and the analysis from them (Mansour et al., 2009) show that the performance of the classifier on the target is in positive correlation with those similar sources.

Very recently, to overcome the so-called negative transfer issue (Rosenstein et al., 2005), MDA methodology has been put forward by leveraging knowledge from multiple sources (Zhang et al., 2015, 2019c). A common problem in MDA is how to reduce the distribution difference between domains (Ghifary et al., 2017). To solve this issue, existing MDA approaches can be simply grouped into two classes (Tao et al., 2019), i.e., classifier-centric learning and feature-centric learning. The former is mainly based on the learning of the source domain classifiers in the target domain to adjust for realizing the implicit adaptation in the target distribution (e.g., Yang et al., 2007; Duan et al., 2012c; Tao et al., 2012; Tommasi et al., 2014), while the latter tries to accomplish the distribution alignment by learning a new representation of the data through a certain transformation (e.g., Wang and Mahadevan, 2011; Ghifary et al., 2017). This article focuses on the research of unsupervised classifier approaches.

In real application scenarios, the classifier-centric MDA scheme usually aims to directly design multiple adaptive source classifiers by merging the multiple distributions’ adaptation *via* feature representation or classifiers with model regularization. Lately, visual recognition works (Mansour et al., 2009; Nie et al., 2010a; Pan et al., 2011; Duan et al., 2012c; Tao et al., 2015, 2017) have proposed a great deal of classifier-centric MDA approaches. One part of classifier-centric MDA research assumes that there are enough number of unlabeled target instances and a large amount of labeled source instances in the training stage. Nevertheless, the remaining part of classifier-centric MDA research holds another hypothesis that only some labeled target instances are accessible in the training stage, which is also called model adaptation in the literature (Yang et al., 2007; Duan et al., 2012b, c; Tao et al., 2015, 2016, 2017; Ghifary et al., 2017). The model adaptation works effectively and efficiently just by exploiting the existing source models pre-trained on relevant but different source domains. Several representative state-of-the-arts include adaptive support vector machines (A-SVM) (Yang et al., 2007) *via* leveraging multiple source classifiers to suit a major target classifier, DA machine (or FastDAM) (Duan et al., 2012c) by employing sparsity regularizations and Laplacian manifold in least squares SVMs (Chai et al., 2016), etc. Recently, we also proposed some different model adaptation strategies (Tao et al., 2015, 2016, 2017) by leveraging the advantages of the low-rank and sparse representation.

### Emotion Recognition

In recent research about affective computing, increasing attentions have been paid on emotion recognition in the community of brain–computer interfaces (BCIs) (Mühl et al., 2014; Chu et al., 2017). An ideal emotion-based BCI can detect the emotional state through spontaneous EEG signals without explicit input from the user (Zhang et al., 2019b) and make a corresponding response to different emotional states. This kind of BCI may enhance the consumer experience in the time of an interactive session. Therefore, different approaches in Zhang et al. (2016, 2017) have been designed to recognize various emotion signals from brain wave. The latest affective BCIs (aBCIs) took machine learning algorithms and depended on a few features with discriminative information (Jenke et al., 2014; Mühl et al., 2014). When recording EEG signals in order to generate a desired target emotion signal, it is necessary to provide users with affective stimulation of specific emotions. In the training/calibration session, the required features and corresponding emotion labels are extracted from EEG signals to train the classifier. In an ongoing BCI session, the feature extractor receives the real-time EEG data and then sends the extracted features to the classifier for real-time affection classification. In this paradigm (Mühl et al., 2014), many researchers have reported pleasing classification performance. However, even if the experimental results are encouraging, the performance of aBCI still could be impacted by some reason. Since the EEG-based emotion signals are different from subject to subject, it is indispensable to train a specific object classifier for the subject of interest. Even in the same subject, the EEG signals are unstable, and the earlier trained classifier may perform poorly in the same subject at a later time. Therefore, in order to maintain a satisfactory classification accuracy, it is necessary to recalibrate frequently.

Domain adaptation method (Judy et al., 2017; Tzeng et al., 2017; Ding et al., 2018) has nearly completely dominated the recent literature of BCI (Jayaram et al., 2016). In aBCI studies, Dolan (2002), Koelstra et al. (2012), Shi et al. (2013), Mühl et al. (2014), Zheng et al. (2015), Zheng and Lu (2015, 2016), Chai et al. (2016, 2017), Lan et al. (2018), Zhong et al. (2020), and search various DA approaches by exploiting the SEED dataset.

## Proposed Framework

### Notations and Definitions

We describe the column vectors and matrices according to the small and capital letters, respectively, in this article. The often utilized symbols are listed in Table 1. The concatenation representation of *k* matrices according to row (horizontally) is like [*A*_1_, *A*_2_, …, *A*_*k*_], and these matrices concatenation operations along a column (vertically) is denoted as [*A*_1_; *A*_2_; …; *A*_*k*_]. The *l*_2,1_−*norm* of *A* is defined as ${||A||}_{2,1}=\sum_{i=1}^{n}{||A_{i,:}||}_{2}=\sum_{i=1}^{n}\sqrt{\sum_{j=1}^{d}A_{ij}^{2}}$, and the trace-norm of *A* is indicated as ${||A||}_{*}=tr\left({\left(AA^{T}\right)}^{\frac{1}{2}}\right)$ (Lotfi and Akbarzadeh, 2014).

**TABLE 1:** Notations and descriptions.

Notations	Descriptions
*n*	Data size
*d*	Feature dimensionality of data
χ	Data space
Γ	Label space
*a* = [*a*_1_, *a*_2_, …, *a*_*d*_]^*T*^ ∈ *R*^*d*^	Feature vector
*A* ∈ *R*^*n*×*d*^	Data matrix
*A*_*i*,*j*_	The (*i*,*j*) entry of *A*
*A*_*i*,:_ and *A*_:,*j*_	The *i*-th row and *j*-th column of *A*
*A*^*T*^ and *a*^*T*^	The transpose of matrix *A* and vector *a*
*tr*(*A*)	The trace of a matrix *A*
$\left\langle A_{1},A_{2}\right\rangle =tr\left(A_{1}^{T}A_{2}\right)$	The inner product of two matrices *A*_1_ and *A*_2_
${||a||}_{p}:={\left(\sum_{i=1}^{d}{||a_{i}||}^{p}\right)}^{1/p}$	The *p*-norm of a vector *a*
${||A||}_{F}=\sqrt{\sum_{i=1}^{n}\sum_{j=1}^{d}A_{i,j}^{2}}$	The Frobenius norm of *A*
*I*_*r*_	Identity matrix of size *r*×*r*
1_*d*_	d-dimensional vector of ones
0_*d*_	d-dimensional vector of zeroes

We mainly focus on the unsupervised MDA based on *S* various sources of *c*-class. Suppose there are *n*_*a*_ (*a* = 1, 2, …, *S*) instances in every source domain, respectively. In the *a*−th source domain, given $X^{a}=\left\{x_{1}^{a},\ldots ,x_{n_{a}}^{a}\right\}\in R^{d\times n_{a}}\in \chi$, and it is a training instances matrix with *c* sub-classes, which are associated with their class labels $Y^{a}={\left[y_{1}^{a},\ldots ,y_{n_{a}}^{a}\right]}^{T}\in R^{n_{a}\times c}\in \Gamma ={\left\{0,1\right\}}^{c\times 1}$, a target domain dataset is denoted as $X^{t}=\left\{x_{1}^{t},x_{2}^{t},\ldots ,x_{m}^{t}\right\}\in R^{d\times n_{t}}\in \chi$, with their pseudo-class labels $Y^{t}={\left[y_{1}^{t},\ldots ,y_{n_{t}}^{t}\right]}^{T}\in R^{n_{t}\times c}\in \Gamma$ obtained from some supervised models (e.g., SVMs) which are trained on the source domain with labeled data. Our ultimate goal is to recognize the ground-truth class of test data $x_{k}^{t}\in X^{t}$, under the conditions that each domain pair *X^a^* and *X^t^* is assumed to be of different marginal and conditional distributions. While we do not need to limit that the instances number in each source domain is identical with that assumed when shaped into the training matrix, for the sake of simplicity, we can extract the same number of training instances from each source domain.

We further denote by $X^{a\left(\bar{l}\right)}\left(\bar{l}=1,\ldots ,c\right)$ the set of samples in *X^a^* with the label $\bar{l}$. Similarly, the sample set in the target domain *X^t^* with the label $\bar{l}$ is defined as $X^{t\left(\bar{l}\right)}$. Note that the true labels of the set $X^{t\left(\bar{l}\right)}$ are unknown. We therefore employ in this work a base classifier, e.g., SVM, to attribute pseudo-labels for the subset in the target domain. For easy expression, we further define the matrix *X*_*a*_ = [*X^a^*, *X^t^*] ∈ R^*d*×*N*^ (*N* = *n*_*t*_ + *n*_*a*_) with its label matrix *Y*_*a*_ = [*Y^a^*, *Y^t^*] in packing both source and target data with respect to the *a−th* source domain.

***Definition 1 (MDA):*** Let Δ = {P^1^, …, P^*S*^} be a set of *S* source domains and P^*t*^∉Δ be a target domain. Denote by $X^{a}={\left\{x_{i}^{a},y_{i}^{a}\right\}}_{i=1}^{n_{a}}\sim {\text{P}}^{a}\left(a=1,\ldots ,S\right)$ the samples drawn from the *a*−th source domains and by $X^{t}={\left\{x_{i}^{t}\right\}}_{i=1}^{n_{t}}∼P^{t}$ the samples drawn from the target domain. The task of MDA is to learn an ensemble function $f_{{\text{P}}^{t}}:𝒳\to \Gamma$ by co-learning multiple classifiers given *X^a^* (*a* = 1, …, *S*) and *X^t^* as the training examples.

***Definition 2 (Multi-source Domain Generalization):*** In this scenario, the target domain is inaccessible in the training stage. Given *S* source domains Δ = {P^1^, …, P^*S*^} and denoted by $X^{a}={\left\{x_{i}^{a},y_{i}^{a}\right\}}_{i=1}^{n_{a}}∼P^{a}$ the samples drawn from the *a*−th source, the task of multi-source generalization is to co-learn multiple adaptive functions $f_{P^{a}}:𝒳\to \Gamma$ only given *X^a^*, ∀*a* = 1, …, *S* as the training examples, which could be well generalized to a certain unseen target domain.

### Problem Statement

In representative MDA, one can use the strategy of acquiring knowledge from multiple auxiliary sources to promote the target task of interest, which is better than learning each source task alone in emotion recognition. That is to say that common knowledge shared by multi-source domains is beneficial to emotion analysis. Moreover, some optimal recognition models have been developed in the latest works for the source domain and/or target domain separately. Furthermore, in these methods, joint multi-source adaption emotion recognition and feature selection has been largely unaddressed, and little or limited efforts have yet been devoted to the utilization of the correlated knowledge among sources.

To solve the above-mentioned issues, we propose in this work a robust multiple-source adaption emotion recognition method based on EEG features. The method utilizes the correlated knowledge among domains and features by joint *l*_2,1_−*norm* and correlation metric regularization and can process high-dimensional, sparse, outliers, and non-i.i.d EEG data at the same time. The designed method has three characteristics, which are integrated into a unified optimization formulation to find an effective emotion recognition model and align the feature distribution between source and target domains: (1) *via* employing the *l*_2,1_−*norm* minimization, a robust loss term is introduced to avoid the influence of noise or outliers in EEG signal, and a sparse regularization term is designed to eliminate over-fitting and a sparse feature subset is selected; (2) based on the designed regression model and the semantic distribution matching between each pair of domains, it not merely provides robustness on loss function but also retains the domain distribution (including local and global) structures and meanwhile maintains a high dependence on the (pseudo)-label knowledge of the source domains and the target domain (Zhang et al., 2020a) so as to obtain preferable generalization performance; and (3) through our constructed metric function of correlation, we can make full use of the correlative information among multiple sources and transfer more discriminative knowledge to the target domain. To implement these properties, in the following part, we will detail the objective formulation of the proposed method.

### General Formulation

In this section, we propose the general formulation of MACI framework underpinned by the robust regression principle and the regularization theory. In particular, our main purpose is to optimize a unified objective function by compromising the following three complementary objectives:

1. Robust multi-source co-regression with feature selection using *l*_2,1_−*norm* minimization, in which the domain label consistency is explicitly maximized through iterative linear label regression.
2. Aligning domain distributions including global statistical distributions and intra-domain semantic distributions or class conditional distributions.
3. Effectively utilizing correlation information among source domains *via* developing an effective correlation metric function.

For the multi-source adaptation emotion recognition of interest, we define the *a*−th (*a* = 1, …, *S*) classifier function as $f_{a}\left(X_{a}\right)=X_{a}^{T}W_{a}$, where *W*_*a*_ is the *a*−th classifier model, and *W*_0_ is certain reference model. Suppose there is a kernel feature map ϕ_*a*_: χ→*H*_*a*_^1^ that projects the training data from the original feature space into certain RKHS (Nie et al., 2010b) *H*_*a*_, the predictor weight *W*_*a*_ can be kernelized. We denote the kernel matrix as ${\left(K_{a}\right)}_{i,j}=\left\langle \phi \left(x_{i}^{a}\right),\phi \left(x_{j}^{a}\right)\right\rangle$, where $x_{i}^{a},x_{j}^{a}\in X_{a}$. We present the empirical kernel map as discussed in Gretton et al. (2009):

$$\begin{array}{cc} \phi_{a}: & \chi \to R^{N},\mathrm{for}\mathrm{linear}\mathrm{kernel}\mathrm{mapping}\\ & x\to {K_{a}\left(\cdot ,x^{a}\right)|}_{x_{1}^{a},x_{2}^{a},\ldots ,x_{N}^{a}}=\left(K_{a}\left(x_{1}^{a},x^{a}\right),\ldots ,K_{a}\left(x_{N}^{a},x^{a}\right)\right), \end{array}$$

fornonlinearkernelmapping

We therefore have kernel matrices *K*_*a*_ = ϕ_*a*_(*X*_*a*_). Hence, the kernelized decision function on *X*_*a*_ (*a* = 1, …, *S*) becomes $f_{a}\left(X_{a}\right)=K_{a}^{T}W_{a}$. We further denote by *W* = [*W*_1_; …; *W*_*S*_] the concatenation matrix.

We then endeavor to find *S* cross-domain models parameterized by ${\left\{W_{a}\right\}}_{a=1}^{S}$ in some empirical RKHSs *via* jointly utilizing correlated knowledge among sources and features. In view of the above-cited objectives, we propose the following general formulation of MACI.

(1) $$\Theta \left(W_{a},F_{a}\right)=R\left(K_{a}^{T}W_{a},Y_{a}\right)+\Omega_{A}\left(X^{a},X^{t}\right)+Cor\left(W\right),$$

where *R*(⋅,⋅) is the robust regression function with feature selection *via l*_2,1_−*norm* minimization, Ω_*A*_(*X^a^*, *X^t^*) is certain distance metric function for aligning the domain distributions, and *Cor*(⋅) is a correlation metric function which is a global regularization term. In the subsequent sections, we focus on designing these components in the general formulation one by one to construct a unified framework.

### Design of Robust Multi-Source Co-regression With Feature Selection

To achieve the first objective mentioned above, one should jointly minimize each source regression loss and implement feature selection, in which the domain label consistency is explicitly maximized, and the data outliers are accounted for to avoid negative transfer. To this end, we first explain a predicted label matrix *F*_*a*_ ∈ R^*N*×*c*^(*a* = 1, …, *S*) into our predictive function (Nie et al., 2010b). The predicted values in this label matrix should satisfy local smoothness and global consistency, i.e., they should preserve the local geometry while fitting in with the true labels (Zhang et al., 2020a). To satisfy these requirements, we present a smooth regularization term on the label geometric structure between each source instance (Nie et al., 2010b; Yan et al., 2006), which is formulated as

$$g\left(F_{a}\right)=\left\{\begin{array}{c} tr\left[{\left(F_{a}-Y_{a}\right)}^{T}\text{Ů}\left(F_{a}-Y_{a}\right)\right]+\alpha tr\left(F_{a}L_{a}{\left(F_{a}\right)}^{T}\right)\\ ||F_{a}^{T}F_{a}=I_{c},{\left(F_{a}\right)}_{i,j}\geq 0 \end{array}\right\},$$

where Ů is a diagonal matrix with Ů_*i*,*i*_ = *ς* (_*ς*_ is a large specified value) if $x_{i}^{a}\in X_{a}$ has a label, Ů_*i*,*i*_ = 0 or else and *a* is a regularization parameter. *L*_*a*_ is the graph Laplacian matrix of the *a*−th source dataset, which is defined as *L*_*a*_ = Λ_*a*_−∏_*a*_, where Λ_*a*_ is a diagonal matrix with (Λ_*a*_)_*i*,*i*_ = ∑_*j*_(∏_*a*_)_*i*,*j*_, and ∏_*a*_ is the weight matrix of the graph, which can be defined as:

$${\left(\prod_{a}\right)}_{i,j}=\left\{ \begin{array}{c} \exp\left(-\gamma_{a}{||x_{i}^{a}-x_{j}^{a}||}^{2}\right),\text{if}x_{i}^{a}\in \delta_{k}\left(x_{j}^{a}\right)\mathrm{or}x_{j}^{a}\in \delta_{k}\left(x_{i}^{a}\right)\mathrm{and}\mathrm{both}\mathrm{have}\mathrm{the}\mathrm{same}\mathrm{labels}\\ \exp\left(-\frac{\gamma_{a}}{{||x_{i}^{a}-x_{j}^{a}||}^{2}}\right),\text{if}x_{i}^{a}\in \delta_{k}\left(x_{j}^{a}\right)\mathrm{or}x_{j}^{a}\in \delta_{k}\left(x_{i}^{a}\right)\mathrm{and}\mathrm{both}\mathrm{have}\mathrm{different}\mathrm{labels}\\ 0,\text{otherwise} \end{array}\right.,$$

where the *_*k*_* nearest neighbors of *x* are assigned to δ_*k*_(*x*), and γ_*a*_ is a hyper-parameter, which can be empirically selected as ${\bar{\theta }}_{a}\sqrt{c}$ by considering the impact of multi-class distribution on the affinity relationship among the domain data, where ${\bar{\theta }}_{a}$ is the square root of the mean norm of _*X_a_*_.

We therefore design the following multi-source sparse co-regression model for meeting the first objective.

(2) $$\begin{array}{c} R\left(W_{a}^{T}\phi \left(X^{a}\right),F_{a}\right)=\sum_{a=1}^{S}\vartheta_{a}^{q_{1}}\left({||K_{a}^{T}W_{a}-F_{a}||}_{2,1}+g\left(F_{a}\right)+\beta {||W_{a}||}_{2,1}\right)\\ s.t.\sum_{a=1}^{S}\vartheta_{a}=1, \end{array}$$

where ϑ = [ϑ_1_, …, ϑ_*a*_]^*T*^ is the weight vector to jointly combine all source regression loss, β is a regularization parameter, and *q*_1_ > 1 is a tunable parameter for avoiding trivial solution. The model (2) is convex, and the *l*_2,1_−*norm* loss function ${||K_{a}^{T}W_{a}-F_{a}||}_{2,1}$ is robust to outliers (Li et al., 2015). In the meantime, the term ||*W*_*a*_||_2,1_ assures that *W*_*a*_ can accomplish feature selection across different domains due to its sparsity. That is, by exploiting the correlation among different features, our approach can jointly evaluate all feature knowledge of source domains and target domain.

#### Design of Domain Distribution Alignment

As a nonparametric distribution discrepancy estimator, MMD (Gretton et al., 2009) was used to compare two distributions by transforming the distributions into a RKHS (Pan et al., 2011; Duan et al., 2012b; Tao et al., 2012; Chen et al., 2013; Long et al., 2014). Let ℱ be a set of functions *f*: 𝒳 → R. The MMD between two domains P and Q is defined as

(3) $$MMD_{ℱ}\left[P,Q\right]:=\underset{f\in ℱ}{\text{sup}}\left(\underset{P}{E}\left[f\left(x\right)\right]-\underset{Q}{E}\left[f\left(x\right)\right]\right).$$

The MMD measures the similarity level between two domains from the side of function class ℱ. To make the MMD a proper regularization for the classifier model *W*_*a*_, we adopt the following the empirical estimation of MMD between *X^a^* and *X^t^*, which is defined as

(4) $$\begin{array}{c} MMD_{e}\left(X^{a},X^{t}\right):={\left\|\frac{1}{n_{a}}\sum_{i=1}^{n_{a}}f_{a}\left(x_{i}^{a}\right)-\frac{1}{n_{t}}\sum_{j=1}^{n_{t}}f_{a}\left(x_{j}^{a}\right)\right\|}_{H}^{2}\\ \;=tr\left(W_{a}^{T}K_{a}M_{a}K_{a}W_{a}\right), \end{array}$$

where ||⋅||_*H*_ is the RKHS norm, ${\left(K_{a}\right)}_{i,j}=<\phi_{a}\left(x_{i}^{a}\right),\phi_{a}\left(x_{j}^{a}\right)>$ with $x_{i}^{a},x_{j}^{a}\in X_{a}$, and

(5) $$M_{i,j}^{a}=\left\{ \begin{array}{c} \begin{array}{ccc} \frac{1}{n_{a}^{2}}, & \text{when} & x_{i}^{a},x_{j}^{a}\in X^{a} \end{array}\\ \begin{array}{ccc} \frac{1}{n_{t}^{2}}, & \text{when} & x_{i}^{a},x_{j}^{a}\in X^{t} \end{array}\\ \begin{array}{ccc} \frac{-1}{n_{a}n_{t}}, & & \text{otherwise} \end{array} \end{array}\right..$$

As for *MMD*_*e*_(*X^a^*,*X^t^*) in Eq. 4, whereas even if there is a perfect domain distribution match, it does not assure the instances from different domains, but the same class of labels will be mapped near the transform space. Lack of semantic consistency will be a major reason for performance degradation. Therefore, we use the following terms to develop a semantically matched MMD (Long et al., 2014):

(6) $$\begin{array}{c} MMD_{CA}\left(X^{a},X^{t}\right)=\sum_{l=1}^{c}MMD_{\varepsilon }\left(X^{a\left(\bar{l}\right)},X^{t\left(\bar{l}\right)}\right)\\ =\sum_{\bar{l}=1}^{c}tr\left(W_{a}^{T}K_{a\left(\bar{l}\right)}M_{a\left(\bar{l}\right)}K_{a\left(\bar{l}\right)}W_{a}\right), \end{array}$$

where $K_{a\left(l\right)}=\phi_{a}\left(\left[X^{a\left(\bar{l}\right)},X^{t\left(\bar{l}\right)}\right]\right)$ with ${\left(K_{a\left(l\right)}\right)}_{i,j}=<\phi_{a}\left(x_{i}^{a\left(\bar{l}\right)}\right),\phi_{a}\left(x_{j}^{a\left(\bar{l}\right)}\right)>$, $x_{i}^{a\left(\bar{l}\right)}\in X^{a\left(\bar{l}\right)}$ and $x_{j}^{t\left(\bar{l}\right)}\in X^{t\left(\bar{l}\right)}$, and

(7) $${\left(M_{a\left(\bar{l}\right)}\right)}_{i,j}=\left\{ \begin{array}{c} \begin{array}{ccc} \frac{1}{n_{a\bar{l}}^{2}}, & \text{when} & x_{i}^{a\left(\bar{l}\right)},x_{j}^{a\left(\bar{l}\right)}\in X^{a\left(\bar{l}\right)} \end{array}\\ \begin{array}{ccc} \frac{1}{m_{\bar{l}}^{2}}, & \text{when} & x_{i}^{a\left(\bar{l}\right)},x_{j}^{a\left(\bar{l}\right)}\in X^{t\left(\bar{l}\right)} \end{array}\\ \begin{array}{ccc} \frac{-1}{n_{a\bar{l}}m_{\bar{l}}}, & & \text{otherwise} \end{array} \end{array}\right..$$

We call Eq. 7 conditional (or semantic) MMD, which explicitly encourages instances from various domains but with the same label to map to the nearest in multi-source subspace. Finally, we suggest that the domain distribution alignment could be approached by learning multiple optimal models such that

(8) $$\begin{array}{c} \Omega_{A}\left(X^{a},X^{t}\right)=MMD_{e}\left(X^{a},X^{t}\right)+MMD_{CA}\left(X^{a},X^{t}\right)\\ =tr\left(W_{a}^{T}K_{a}M_{a}K_{a}W_{a}\right)+\sum_{l=1}^{c}tr\left(W_{a}^{T}K_{a\left(\bar{l}\right)}M_{a\left(\bar{l}\right)}K_{a\left(\bar{l}\right)}W_{a}\right)\\ =\sum_{\bar{l}=0}^{c}tr\left(W_{a}^{T}C_{a\left(\bar{l}\right)}W_{a}\right)=tr\left(W_{a}^{T}\sum_{\bar{l}=0}^{c}C_{a\left(\bar{l}\right)}W_{a}\right), \end{array}$$

where *C*_*a*(0)_ = *K*_*a*_*M*_*a*_*K*_*a*_ and $C_{a\left(\bar{l}\right)}=K_{a\left(\bar{l}\right)}M_{a\left(\bar{l}\right)}K_{a\left(\bar{l}\right)}\left(\bar{l}=1,\ldots ,c\right)$. Let $C_{a}=\sum_{\bar{l}=0}^{c}C_{a\left(\bar{l}\right)}$, then we have $\Omega_{A}\left(X^{a},X^{t}\right)=tr\left(W_{a}^{T}C_{a}W_{a}\right)$.

#### Design of Correlation Metric Function

As we know, a commonly used strategy in extant classifier-centric adaptation methods (Duan et al., 2012c; Tommasi et al., 2014) is to directly match the discriminant models between different domains, which is defined as:

***Definition 3 (model discriminant discrepancy, MDD):*** Let 𝒲 be a set of function parameters 𝒲: 𝒳 → R. The model discriminant discrepancy between domains P and Q is defined as

$$MDD_{𝒲}\left[P,Q\right]:=\underset{W_{P},W_{Q}\in 𝒲}{\text{sup}}{||W_{P}-W_{Q}||}_{F}^{2}.$$

It may be difficult to push these two models respectively learnt from different domains when the distribution discrepancy between them is large. In our correlation metric function, we instead aim to guarantee each source model to be aligned with a global reference matrix *W*_0_ so as to enable different source models to share the common knowledge for effectively utilizing correlation information among source domains. In essence, *W*_0_ builds a transformation among source domains so that knowledge of one source can be used to another. They yield the following model alignment function (MAF):

***Definition 4 (model alignment function):*** Given *S* domains ${\left\{X^{a}\right\}}_{a=1}^{S}$ on 𝒳, we can think of the classification model set ${\left\{W^{a}\right\}}_{a=1}^{S}$ in some latent spaces. Their MAF is then defined as

$$\Psi \left({\left\{W_{a}\right\}}_{a=1}^{S}\right)=\sum_{a=1}^{S}\eta_{a}{||W_{a}-W_{0}||}_{F}^{2}$$

where η = [η_1_, …, η_*S*_]^*T*^ is a weight vector for discriminatively selecting different source knowledge with $\sum_{a=1}^{S}\eta_{a}=1$, and *W*_0_ is certain shared (common) discriminant model among these domains (Zhang et al., 2019a).

In essence, the MAF measures the similarity between two domain classifiers by the classification model. The next theorem is about from MAF to MDD between two domains.

***Theorem 1 (MAF bounds MDD):*** The (squared) maximum discriminant discrepancy between domains P and Q on 𝒳 is upper-bounded by their MAF with η_P_ = η_Q_ = η_Δ_ :

$$\eta_{\Delta }MDD_{𝒲}^{2}\left[P,Q\right]\leq \Psi \left(\left\{W_{P},W_{Q}\right\}\right)$$

where 𝒲 = {*W*: 𝒳 → R|*W* is the classifier model} and *W*_P_, *W*_Q_ ∈ 𝒲. Specially if 𝒲 is induced by a characteristic kernel on 𝒳, then Ψ ({*W*_P_, *W*_Q_}) = 0 if and only if P = Q.

**Proof.** By definition 4 and triangle inequality theorem,

$$\begin{array}{c} \Psi \left(\left\{W_{P},W_{Q}\right\}\right)\\ =\eta_{P}{||W_{P}-W_{0}||}_{F}^{2}+\eta_{Q}{||W_{P}-W_{0}||}_{F}^{2}\\ =\eta_{\Delta }\left({||W_{P}-W_{0}||}_{F}^{2}+{||W_{Q}-W_{0}||}_{F}^{2}\right)\\ \geq \eta_{\Delta }{||\left(W_{P}-W_{0}\right)-\left(W_{Q}-W_{0}\right)||}_{F}^{2}\\ =\eta_{\Delta }{||W_{P}-W_{Q}||}_{F}^{2}=\eta_{\Delta }MDD_{𝒲}^{2}\left[P,Q\right], \end{array}$$

that is, Ψ({*W*_P_, *W*_Q_}) bounds $MDD_{𝒲}^{2}\left[P,Q\right]$. If 𝒲 is induced by some characteristic kernel on 𝒳, then Ψ({*W*_P_, *W*_Q_}) = 0 if and only if P = Q, which can be concluded from the result of Theorem 2.2 of Gretton et al. (2009).

Theorem 1 also indicates that the MAF is an effective metric method if the kernel on 𝒳 is characteristic (Gretton et al., 2009).

***Theorem 2:*** In particular, if *W*_0_ = η_P_*W*_P_ + η_Q_*W*_Q_ in MAF, we obtain

$$\Psi \left(\left\{W_{P},W_{Q}\right\}\right)=\eta_{P}\eta_{Q}MDD_{𝒲}^{2}\left[P,Q\right]\leq \frac{1}{4}MDD_{𝒲}^{2}\left[P,Q\right].$$

**Proof.** By η_*P*_ + η_*Q*_ = 1, we have

$$\begin{array}{c} \Psi \left(\left\{W_{P},W_{Q}\right\}\right)\\ =\eta_{P}{||W_{P}-\left(\eta_{P}W_{P}+\eta_{Q}W_{Q}\right)||}_{F}^{2}\\ +\eta_{Q}{||W_{Q}-\left(\eta_{P}W_{P}+\eta_{Q}W_{Q}\right)||}_{F}^{2}\\ =\eta_{P}{||\eta_{Q}W_{P}-\eta_{Q}W_{Q}||}_{F}^{2}+\eta_{Q}{||\eta_{P}W_{P}-\eta_{P}W_{Q}||}_{F}^{2}\\ =\left(\eta_{P}\eta_{Q}^{2}+\eta_{Q}\eta_{P}^{2}\right){||W_{P}-W_{Q}||}_{F}^{2}=\eta_{P}\eta_{Q}MDD_{𝒲}^{2}\left[P,Q\right]\\ \leq \frac{1}{4}MDD_{𝒲}^{2}\left[P,Q\right], \end{array}$$

where the last inequality follows after observing that η_P_η_Q_ ≤ 1/4 with the equality holding when η_P_ = η_Q_ = 1/2.

For achieving the third target of ours, the correlation metric function therefore was designed as follows:

(9) $$Cor\left(W\right)=\sum_{a=1}^{S}\eta_{a}^{q_{2}}{||W_{a}-W_{0}||}_{F}^{2}+\frac{\lambda }{2}{||W||}_{*},$$

where *q*_2_ > 1 is another tunable parameter to avoid a trivial solution. The regularization term ||*W*||_*_ in Eq. 9 enables different projection functions ${\left\{W_{a}\right\}}_{a=1}^{S}$ to share common information/parts through models of sources. Thus, the knowledge from multiple sources can be further shifted from one by one source domain.

### Final Formulation

By using empirical kernel function and integrating Eqs 2, 8, and 9, we therefore propose the following unified framework to implement the combination of feature selection and domain adaptive learning by utilizing the correlated knowledge across multi-sources and features.

(10) $$\begin{array}{c} \min_{W_{a},F_{a},\vartheta_{a},\eta_{a}}\sum_{a=1}^{S}(\vartheta_{a}^{q_{1}}\left(||K_{a}^{T}W_{a}-F_{a}|{|}_{2,1}+tr\left(W_{a}^{T}C_{a}W_{a}\right)\right)\\ +g\left(F_{a}\right)+\beta ||W_{a}|{|}_{2,1})+\sum {}_{a=1}{}^{S}\eta {}_{a}{}^{q_{2}}||W{}_{a}-W{}_{0}||{}_{F}{}^{2}+\frac{\lambda }{2}||W||{}_{*}\\ s.t.\sum_{a=1}^{S}\vartheta_{a}=\sum_{a=1}^{S}\eta_{a}=1. \end{array}$$

Note that, in Eq. 10, the *l*_2,1_−*norm* loss function makes the model effectively robust to noises or outliers from domains. In addition, after *l*_2,1_−*norm* regularization is added to *W*_*a*_, many rows in *W*_*a*_(*a* = 1, …, *S*) become zero. Therefore, the characteristics corresponding to these rows with zeros are not significant for the target task learning. Therefore, for acquiring competitive performance, we can select features from the original domain data. Similarly (Nie et al., 2010a), we sort the rows in *W*_*a*_ by descending sequence in light of each row of values in *l*_2_−*norm* and followed by selecting the top rows as the feature selection outcome.

***Remark 1:*** Our proposed method (10) has some competitions in universalization and efficiency. Firstly, in order to deal with the change of feature dimensions and types in among source domains easily, we learn a separate classification model for each independent domain pair (i.e., one source domain and one target domain). Then, for dealing with more heterogeneous sources, the algorithm is easy to extend. Moreover, in order to improve the speed of the algorithm, the redundant and irrelevant knowledge in the original features is thrown away before classification through sparse feature selection method, and then the classification models are learned. Furthermore, joint *l*_2,1_−*norm* and trace-norm minimization can be used to express well the together learning of feature selection and classification models so as to assure that the common subspace in the multi-domain can be extracted.

## Algorithm

In this section, we first give an iterative approach to optimize the objective function (10) followed by its complexity and classification function. Although matrix completion is realized by an alike optimization method from Yang et al. (2013), we focus on the other issue, that is, joint optimization of trace norm and *l*_2,1_−*norm*. We then further present an effective and valuable extension to domain generalization when the target domain is inaccessible.

### Algorithm Optimization

According to Nie et al. (2010a), the derivative of *tr*(T̊^*T*^*Q*T̊) is equal to the derivative of ||T̊||_2,1_, i.e., 2*tr*(T̊^*T*^*Q*T̊) = ||T̊||_2,1_, where *Q* is a diagonal matrix and its *i*−th diagonal value is $Q_{ii}=\frac{1}{2{||{\text{T̊}}_{i,:}||}_{2}}$, and if T̊_*i*,:_ = 0, we can let $Q_{ii}=\frac{1}{2{||{\text{T̊}}_{i,:}||}_{2}+\varepsilon }$, where ε is a very small given value. Hence, we can farther transform Eq. 10 into Eq. 11:

(11) $$\begin{array}{c} \min_{W_{a},F_{a},\vartheta_{a},\eta_{a}}\sum_{a=1}^{S}(\vartheta_{a}^{q_{1}}tr\left(T_{a}^{T}Z_{a}T_{a}\right)+\vartheta_{a}^{q_{1}}tr\left(W_{a}^{T}C_{a}W_{a}\right)\\ +g\left(F_{a}\right)+\beta tr\left(W_{a}^{T}G_{a}W_{a}\right))\\ +\sum_{a=1}^{S}\eta_{a}^{q_{2}}{||W_{a}-W_{0}||}_{F}^{2}+\frac{\lambda }{2}tr\left(W^{T}{\left(WW^{T}\right)}^{-\frac{1}{2}}W\right)\\ s.t.\sum_{a=1}^{S}\vartheta_{a}=\sum_{a=1}^{S}\eta_{a}=1, \end{array}$$

where $S_{a}={\left[\underset{a-1}{\underbrace{0_{r},\ldots ,0_{r}}},I_{r},\underset{S-a}{\underbrace{0_{r},\ldots ,0_{r}}}\right]}^{T}$, the diagonal matrix *G*_*a*_ is based on *W*_*a*_, where the *k*−th element is equal to ${\left(G_{a}\right)}_{kk}=\frac{1}{2{||{\left(W_{a}\right)}_{k,:}||}_{2}}$, the diagonal matrix *Z*_*a*_ is based on $T_{a}=\sqrt{\vartheta_{a}^{q_{1}}}\left(K_{a}^{T}W_{a}-F_{a}\right)$, and where the *k*−th entry is computed by ${\left(Z_{a}\right)}_{kk}=\frac{1}{2{||{\left(T_{a}\right)}_{k,:}||}_{2}}$. By taking the derivative of Eq. (11) in reference to *W*_0_ and equaling to zero, we obtain:

(12) $$W_{0}=W\eta_{0},$$

where $\eta_{0}=\left[{\tilde{\eta }}_{1}I_{r};\ldots ;{\tilde{\eta }}_{S}I_{r}\right]$ with ${\tilde{\eta }}_{a}=\eta_{a}^{q_{2}}/\sum_{a=1}^{S}\eta_{a}^{q_{2}}\left(a=1,\ldots ,S\right)$. Substituting Eq. 12 into Eq. 11, we have

(13) $$\begin{array}{c} \min_{W_{a},F_{a},\vartheta_{a},\eta_{a}}\sum_{a=1}^{S}\{tr\left(T_{a}^{T}Z_{a}T_{a}\right)+\vartheta_{a}^{q_{1}}tr\left(W_{a}^{T}C_{a}W_{a}\right)\\ +g\left(F_{a}\right)+\beta tr\left(W_{a}^{T}G_{a}W_{a}\right)\}\\ +\sum_{a=1}^{S}\eta_{a}^{q_{2}}{||WS_{a}-W\eta_{0}||}_{F}^{2}+\frac{\lambda }{2}tr\left(W^{T}{\left(WW^{T}\right)}^{-\frac{1}{2}}W\right). \end{array}$$

Note that the sub-gradient matrices *Z*_*a*_ and *G*_*a*_ in Eq. 13 are dependent on the matrices *W*_*a*_ and *F*_*a*_, which are also unknown beforehand. Consequently, the objective function in Eq. 13 is a multi-variable optimization problem involving the variables *W*_*a*_, *F*_*a*_, ϑ_*a*_, η_*a*_. Since optimizing these variables simultaneously is difficult, we exploit the alternating iterative strategy to update one variable iteratively while the other variable(s) is(are) fixed. Therefore, the problem in Eq. 13 can be decomposed into four sets of convex sub-problems. We aim to find the optimal solution to each sub-problem alternatively and iteratively so that the objective function in Eq. 13 would converge to a local optimal solution. By initializing *W*_*a*_ and *F*_*a*_, thus initializing *Z*_*a*_ and *G*_*a*_, then we can start the iterations.

#### Optimize *W*_*a*_ and *F*_*a*_ by Fixing ϑ and η

By solving the derivative of Eq. 13 in reference to *W*_*a*_ and equaling to zero, we obtain:

$$W_{a}=\vartheta_{a}^{q_{1}}{\left(\vartheta_{a}^{q_{1}}C_{a}+\vartheta_{a}^{q_{1}}K_{a}Z_{a}K_{a}+\beta G_{a}+\Omega +\lambda U\right)}^{-1},$$

(14) $$K_{a}Z_{a}F_{a}=E_{a}F_{a}$$

where

(15) $$U=\frac{1}{2}{\left(WW^{T}\right)}^{-\frac{1}{2}},$$

(16) $$\Omega =\sum_{a=1}^{S}\left(\eta_{a}^{q_{2}}{\left(S_{a}-\eta_{0}\right)}^{T}\left(S_{a}-\eta_{0}\right)\right),$$

and

(17) $$E_{a}=\vartheta_{a}^{q_{1}}{\left(\vartheta_{a}^{q_{1}}C_{a}+\vartheta_{a}^{q_{1}}K_{a}Z_{a}K_{a}+\beta G_{a}+\Omega +\lambda U\right)}^{-1}K_{a}Z_{a}.$$

Plugging Eq. 14 into Eq. 13, by mathematical calculating, we can get:

(18) $$tr\left(F_{a}^{T}N_{a}F_{a}\right)+\alpha tr\left(F_{a}L_{a}{\left(F_{a}\right)}^{T}\right)+tr\left[{\left(F_{a}-Y_{a}\right)}^{T}\text{Ů}\left(F_{a}-Y_{a}\right)\right],$$

where

$$N_{a}=E_{a}^{T}\left(C_{a}+\beta G_{a}+\lambda U\right)E_{a}+\alpha L_{a}+{\left(K_{a}^{T}E_{a}-I_{n}\right)}^{T}$$

$$Z_{a}\left(K_{a}^{T}E_{a}-I_{n}\right).$$

Lastly, substituting the optimal solution of the other variables into Eq. 18 to update *F*_*a*_, the constraints $F_{a}^{T}F_{a}=I_{c}$ should be added additionally, and (*F*_*a*_)_*ij*_ ≥ 0. Then, we can get the objective function in reference to *F*_*a*_:

(19) $$\begin{array}{c} \Theta \left(F_{a}\right)=\min_{F_{a}}tr\left(F_{a}^{T}\left(N_{a}+\alpha L_{a}\right)F_{a}\right)+\\ tr\left[{\left(F_{a}-Y_{a}\right)}^{T}\text{Ů}\left(F_{a}-Y_{a}\right)\right]+\frac{\zeta }{2}{||F_{a}^{T}F_{a}-I_{c}||}_{F}^{2}+tr\left(\theta F_{a}^{T}\right), \end{array}$$

where ζ ^2^ and θ are balance arguments. By solving the derivative of Eq. 19 in reference to *F*_*i*,*j*_ and equaling to zero and exploiting the K.K.T. with constraint term θ_*ij*_*F*_*i*,*j*_ = 0, we can get:

(20) $$\begin{array}{c} \frac{\partial \Theta \left(F_{a}\right)}{\partial F_{a}}=\left(N_{a}+\alpha L_{a}+\text{Ů}\right)F_{a}-\text{Ů}Y_{a}+\zeta F_{a}\left(F_{a}^{T}F_{a}-I_{c}\right)\\ +\theta /2=0\\ \Rightarrow {\left(F_{a}\right)}_{i,j}\leftarrow {\left(F_{a}\right)}_{i,j}\frac{{\left(\text{Ů}Y_{a}+\zeta F_{a}\right)}_{i,j}}{{\left(\zeta F_{a}F_{a}^{T}F_{a}+\left(N_{a}+\alpha L_{a}+\text{Ů}\right)F_{a}\right)}_{i,j}}. \end{array}$$

#### Optimize ϑ_*a*_ by Fixing *W*_*a*_, *F*_*a*_, and η_*a*_

In this situation, the issue in Eq. 13 changes to a small problem as follows:

(21) $$\min_{\vartheta_{a}\geq 0,\vartheta_{a}^{T}1=1}\sum_{a=1}^{S}\left\{\vartheta_{a}^{q_{1}}tr\left(T_{a}^{T}Z_{a}T_{a}\right)+\vartheta_{a}^{q_{1}}tr\left(W_{a}^{T}C_{a}W_{a}\right)\right\}.$$

Let $g_{a}=tr\left(T_{a}^{T}Z_{a}T_{a}\right)+tr\left(W_{a}^{T}C_{a}W_{a}\right)$, the Lagrange function of Eq. 21 is

(22) $$ℑ\left(\vartheta_{a},\phi \right)=\sum_{a=1}^{S}\vartheta_{a}^{q_{1}}g_{a}-\phi \left(\sum_{a=1}^{S}\vartheta_{a}-1\right).$$

Setting the derivative of ℑ⁡(ϑ_*a*_, ϕ) with respect to ϑ_*a*_ is equivalent to 0, and we can obtain:

(23) $$\vartheta_{a}={\left(\phi /\left(q_{1}g_{a}\right)\right)}^{\frac{1}{q_{1}-1}}.$$

Substituting Eq. 23 into the constraint $\sum_{a=1}^{S}\vartheta_{a}=1$, we obtain

(24) $$\vartheta_{a}={\left(g_{a}\right)}^{1/\left(1-q_{1}\right)}/\sum_{a=1}^{S}{\left(g_{a}\right)}^{1/\left(1-q_{1}\right)}.$$

#### Optimize η_*a*_ by Fixing *W*_*a*_, *F*_*a*_, and ϑ_*a*_

By fixing *W*_*a*_, *F*_*a*_, and ϑ_*a*_, the problem in Eq. 13 then becomes the following sub-problem:

(25) $$\min_{\eta_{a}\geq 0,\eta_{a}^{T}1=1}\sum_{a=1}^{S}\eta_{a}^{q_{2}}{||WS_{a}-W\eta_{0}||}_{F}^{2}.$$

Let $h_{a}={||WS_{a}-W\eta_{0}||}_{F}^{2}$, the Lagrange function of Eq. 25 is

(26) $$ℑ\left(\eta_{a},\rho \right)=\sum_{a=1}^{S}\eta_{a}^{q_{2}}h_{a}-\rho \left(\sum_{a=1}^{S}\eta_{a}-1\right).$$

Setting the derivative of ℑ⁡(η_*a*_, ρ) in reference to η_*a*_ is equivalent to 0, and we then get:

(27) $$\eta_{a}={\left(\rho /\left(q_{2}h_{a}\right)\right)}^{\frac{1}{q_{2}-1}}.$$

Substituting Eq. 27 into the constraint $\sum_{a=1}^{S}\eta_{a}=1$, we obtain:

(28) $$\eta_{a}={\left(h_{a}\right)}^{1/\left(1-q_{2}\right)}/\sum_{a=1}^{S}{\left(h_{a}\right)}^{1/\left(1-q_{2}\right)}.$$

#### Overall Procedure

In this sub-section, we finally report the whole optimization process of MACI in Algorithm 1, where a window-based breaking criterion is employed to better obtain the convergence of the algorithm (Zhang et al., 2019a). Concretely speaking, defining a window size ℏ, we compute *ς* = ||*Max*Θ_*itr*_−*Min*Θ_*itr*_||/*Max*Θ_*itr*_ in *itr*−th iteration, where Θ_*itr*_ = {*Obj*_*itr*−ℏ + 1_, …, *Obj*_*itr*_} represents the set of historical target values in the window. When *ς* is less than a given threshold ε, that is *ς* < ε, the algorithm stops iterating. In our experiments, we set ε = 10^−5^ and ℏ = 6 empirically without losing statistical performance. We will discuss in section “Convergence” why Algorithm 1 is convergent.

### Computational Complexity

In this subsection, we give a formal analysis about the computational complexity of several main components in Algorithm 1 using the big *O* notation. Firstly, the construction of the *k*−*NN* graph and computing of the kernel matrix ${\left\{K_{i}\right\}}_{i=1}^{S}$, respectively, need computational cost $O\left(Sdn_{a}^{2}\right)$ and *O*(*SdN*^2^). Then, the optimization proceeds according to step by step iteratively. The cost for computing *F*_*a*_ is $O\left(3n_{a}^{3}+n_{a}^{2}c\right)$. After *F*_*a*_ was updated, computing *W*_*a*_ would cost *O*(*n*_*a*_*c*^2^ + *dc*^2^). In a word, the whole calculating cost is $O\left(\ell S\left(3n_{a}^{3}+n_{a}^{2}c+n_{a}c^{2}+dc^{2}\right)+Sdn_{a}^{2}+SdN^{2}\right)$. We assume that all Laplacian matrixes ${\left\{L_{i}\right\}}_{i=1}^{S}$ can be pre-calculated before the iterative optimization of Algorithm 1, and multi-kernel can be pre-calculated and put into memory before training. Thus, Algorithm 1 is effective and efficient computationally.

### Target Classification

The datasets of the unlabeled samples from the target domain are defined as $K_{u}^{t}={\left\{\left(k_{j}^{t}\right)\right\}}_{j=1}^{n_{t}}$. In the unsupervised DA learning case, one can predict a target class using the classification model *W*_0_. Specifically, one may use $\arg\max_{1\leq j\leq c}{\left(\Gamma_{u}^{t}\right)}_{j}$ to classify a test sample $k_{u}^{t}\in K_{u}^{t}$ into one of the *c* target classes, where $\Gamma_{u}^{t}={\left(W_{0}\right)}^{T}k_{u}^{t}$.

In our multi-source adaptation framework, nevertheless, another voting method defined as “sum” can be deduced, that is, once ${\left\{W_{a}^{s}\right\}}_{a=1}^{S}$ are obtained, for a test data $k_{u}^{t}\in K_{u}^{t}$, we can learn its label vector $\Gamma_{u}^{t}$ by minimizing the residue between $\Gamma_{u}^{t}$ and the projected vector of each source model:

(29) $$\min_{\Gamma_{u}^{t}}\sum_{a=1}^{S}\vartheta_{a}{||{\left(k_{u}^{t}\right)}^{T}W_{a}^{s}-\Gamma_{u}^{t}||}_{2}^{2}.$$

**Table:** 

**Algorithm 1. Multiple sources adaptation learning by utilizing correlation knowledge.**
Input: Source datasets ${\left\{X_{i}^{s}\right\}}_{i=1}^{S}$, ${\left\{L_{i}\right\}}_{i=1}^{S}$, target dataset *X^t^*, and parameters α, β, and λ, the maximal iteration number ℓ. Output: Converged projection matrices ${\left\{W_{i}\right\}}_{i=1}^{S}$, and matrices ${\left\{F_{i}\right\}}_{i=1}^{S}$ and *W*_0_. Initialization: Set *itr* = 0, and initialize ${\left\{W_{i}^{itr}\right\}}_{i=0}^{S}$ randomly. Let $W^{itr}=\left[W_{1}^{itr},\ldots ,W_{S}^{itr}\right]$; 1: for *i* = 1 to *S* do { Compute matrix $M_{i}^{itr}$ and $M_{i\left(l\right)}^{itr}$, and $K_{i}^{itr}$ and $K_{i\left(l\right)}^{itr}$ with empirical kernel mapping, thus computing $C_{i}^{itr}=\sum_{l=0}^{c}C_{i\left(l\right)}^{itr}$ by $C_{i\left(0\right)}^{itr}=K_{i}^{itr}M_{i}^{itr}K_{i}^{itr}$ and $C_{i\left(l\right)}^{itr}=K_{i\left(l\right)}^{itr}M_{i\left(l\right)}^{itr}K_{i\left(l\right)}^{itr}$, *l* = 1, …, *c*; Compute $\eta_{i}^{itr}$ by Eq. 28, and then construct matrix η^*itr*^ and $\eta_{0}^{itr}=\left[\eta_{1}^{itr}I_{r};\ldots ;\eta_{S}^{itr}I_{r}\right]$; Compute $F_{i}^{itr}=K_{i}^{T}W_{i}^{itr}$; } 2: repeat { Compute $W_{0}^{itr}$ by (12); Compute the diagonal matrix *U*^*itr*^ by (15); Compute the matrix Ω^*itr*^ by (16); set *i* = 1; repeat { Compute $G_{i}^{itr}$ with respect to $W_{i}^{itr}$; Compute $Z_{i}^{itr}$ with respect to $K_{i}^{T}W_{i}^{itr}-F_{i}^{itr}$; Compute $g_{i}^{itr}=tr\left({\left(K_{i}^{T}W_{i}^{itr}-F_{i}^{itr}\right)}^{T}Z_{i}\left(K_{i}^{T}W_{i}^{itr}-F_{i}^{itr}\right)\right)\left[cpsbreak\right]+tr\left({\left(W_{i}^{itr}\right)}^{T}C_{i}^{itr}W_{i}^{itr}\right)$; Compute $\vartheta_{i}^{itr}$ according to Eq. 24, and then construct ϑ^*itr*^; Compute the matrix $E_{i}^{itr}$ by Eq. 17, and then $N_{i}^{itr}$ by Eq. 19; Compute $F_{i}^{itr}$ according to Eq. 20; Compute $W_{i}^{itr}$ according to Eq. 14; *i* = *i* + 1; } until *i* > *S* Update $W_{i}^{itr+1}=W_{i}^{itr}$s.t.*i* = 1,..,*S*; Update $F_{i}^{itr+1}=F_{i}^{itr}$ according to (20) s.t.*i* = 1,..,*S*; Update $\vartheta_{i}^{itr+1}$ according to (24) s.t.*i* = 1,..,*S*; Update $\eta_{i}^{itr+1}$ according to (27) s.t.*i* = 1,..,*S*; Update $W_{0}^{itr+1}$ according to (12); Let *itr* = *itr* + 1; }until *itr* > ℓ or *ς* < 10^−5^ 3: return ${\left\{W_{a}\right\}}_{a=0}^{S}$ and ${\left\{F_{i}\right\}}_{i=1}^{S}$.

The result of Eq. 29 can be acquired according to the constraint term $\sum_{a=1}^{S}\vartheta_{a}=1$:

(30) $$\Gamma_{u}^{t}=\sum_{a=1}^{S}\vartheta_{a}{\left(k_{u}^{t}\right)}^{T}W_{a}^{s}.$$

Once $\Gamma_{u}^{t}$ is computed by using Eq. 30, we then use $\arg\max_{1\leq j\leq c}{\left(\Gamma_{u}^{t}\right)}_{j}$ to determine the class for this test data.

## Algorithm Analysis

### Convergence

We start with the next two lemmas and then demonstrate that the alternant optimization process, namely, step 2 in Algorithm 1, in the optimization issue of the Eq. 10, the optimal solution of ${\left\{W_{i}\right\}}_{i=1}^{S}$ converges.

***Lemma 1.*** (Nie et al., 2010a) There are any two values *V*_1_,*V*_2_ ∈ R^*d*^, and they are not equal to zero; we can get the inequality as:

(31) $${||V_{1}||}_{2}-\frac{{||V_{1}||}_{2}^{2}}{2{||V_{2}||}_{2}}\leq {||V_{2}||}_{2}-\frac{{||V_{2}||}_{2}^{2}}{2{||V_{2}||}_{2}}.$$

***Lemma 2.*** (Nie et al., 2010a) For any invertible matrices P̊ and Q̊, the following inequality holds:

(32) $$\frac{1}{2}tr\left({\text{P̊Q̊}}^{-\frac{1}{2}}\right)-tr\left({\text{P̊}}^{\frac{1}{2}}\right)\geq \frac{1}{2}tr\left({\text{Q̊Q̊}}^{-\frac{1}{2}}\right)-tr\left({\text{Q̊}}^{\frac{1}{2}}\right).$$

Then, the iterative method designed in Algorithm 1 can converge to the optimal solution, which will be proved in the next theorem.

***Theorem 3:*** In each iteration of Algorithm 1, the objective function of issue in Eq. 10 will be monotonically decreasing and finally will converge to the optimal solution of the issue.

**Proof.** For easy description, we define the updated *W*_*i*_ and *F*_*i*_ in the iteration τ as $W_{i}^{\tau }$ and $F_{i}^{\tau }\left(i=1,\ldots ,S\right)$ separately. The updating from step 2 of Algorithm 1 is equivalent to the optimum of the next problem:

$$\begin{array}{c} \min_{W_{i},F_{i},\vartheta_{i},\eta_{i}}\sum_{i=1}^{S}\{\vartheta_{i}^{q_{1}}[tr\left({\left(K_{i}^{T}W_{i}-F_{i}\right)}^{T}Z_{i}\left(K_{i}^{T}W_{i}-F_{i}\right)\right)\\ +tr\left(W_{i}^{T}C_{i}W_{i}\right)]+\alpha g\left(F_{i}\right)+\beta tr\left(W_{i}^{T}G_{i}W_{i}\right)\}\\ +tr\left(W^{T}\Omega W\right)+\lambda tr\left(W^{T}UW\right) \end{array}$$

Following the expressions of *Z*_*i*_, *G*_*i*_, and *U*, then we can get:

(33) $$\begin{array}{c} \sum_{i=1}^{S}\left\{\begin{array}{c} tr\left({\left(W_{i}^{\tau +1}\right)}^{T}C_{i}W_{i}^{\tau +1}\right)+\alpha g\left(F_{i}^{\tau +1}\right)\\ +\vartheta_{i}^{q_{1}}\sum_{j=1}^{n}\frac{{||Z_{i}{\left(j,:\right)}^{\tau +1}||}_{2}^{2}}{{||Z_{i}{\left(j,:\right)}^{\tau }||}_{2}}+\beta \sum_{j=1}^{n}\frac{{||W_{i}{\left(j,:\right)}^{\tau +1}||}_{2}^{2}}{{||W_{i}{\left(j,:\right)}^{\tau }||}_{2}} \end{array}\right\}\\ +tr\left({\left(W^{\tau +1}\right)}^{T}\Omega^{\tau +1}W^{\tau +1}\right)+\lambda tr\left({\left(W^{\tau +1}\right)}^{T}U^{\tau }W^{\tau +1}\right)\\ \leq \sum_{i=1}^{S}\left\{\begin{array}{c} tr\left({\left(W_{i}^{\tau }\right)}^{T}C_{i}W_{i}^{\tau }\right)+\alpha g\left(F_{i}^{\tau }\right)\\ +\vartheta_{i}^{q_{1}}\sum_{j=1}^{n}\frac{{||Z_{i}{\left(j,:\right)}^{\tau }||}_{2}^{2}}{{||Z_{i}{\left(j,:\right)}^{\tau }||}_{2}}+\beta \sum_{j=1}^{n}\frac{{||W_{i}{\left(j,:\right)}^{\tau }||}_{2}^{2}}{{||W_{i}{\left(j,:\right)}^{\tau }||}_{2}} \end{array}\right\}\\ +tr\left({\left(W^{\tau }\right)}^{T}\Omega^{\tau }W^{\tau }\right)+\lambda tr\left({\left(W^{\tau }\right)}^{T}U^{\tau }W^{\tau }\right). \end{array}$$

We can have the next inequality by Lemma 1:

(34) $$\begin{array}{c} \sum_{j=1}^{n}\left({\left\|{\left(W_{i}\right)}_{j,:}^{\tau +1}\right\|}_{2}-\frac{{\left\|{\left(W_{i}\right)}_{j,:}^{\tau +1}\right\|}_{2}^{2}}{2{\left\|{\left(W_{i}\right)}_{j,:}^{\tau }\right\|}_{2}}\right)\\ \;\leq \sum_{j=1}^{n}\left({\left\|{\left(W_{i}\right)}_{j,:}^{\tau }\right\|}_{2}-\frac{{\left\|{\left(W_{i}\right)}_{j,:}^{\tau }\right\|}_{2}^{2}}{2{\left\|{\left(W_{i}\right)}_{j,:}^{\tau }\right\|}_{2}}\right). \end{array}$$

Therefore, we have

(35) $$\begin{array}{c} \sum_{i=1}^{S}\left\{\begin{array}{c} tr\left({\left(W_{i}^{\tau +1}\right)}^{T}C_{i}W_{i}^{\tau +1}\right)+\alpha g\left(F_{i}^{\tau +1}\right)\\ +\vartheta_{i}^{q_{1}}\sum_{j=1}^{n}{||{\left(Z_{i}\right)}_{j,:}^{\tau +1}||}_{2}+\beta \sum_{j=1}^{n}{||{\left(W_{i}\right)}_{j,:}^{\tau +1}||}_{2} \end{array}\right\}\\ +tr\left({\left(W^{\tau +1}\right)}^{T}\Omega^{\tau +1}W^{\tau +1}\right)+\lambda tr\left({\left(W^{\tau +1}\right)}^{T}U^{\tau +1}W^{\tau +1}\right)\\ \leq \sum_{i=1}^{S}\left\{\begin{array}{c} tr\left({\left(W_{i}^{\tau }\right)}^{T}C_{i}W_{i}^{\tau }\right)+\alpha g\left(F_{i}^{\tau }\right)\\ +\vartheta_{i}^{q_{1}}\sum_{j=1}^{n}{||{\left(Z_{i}\right)}_{j,:}^{\tau }||}_{2}+\beta \sum_{j=1}^{n}{||{\left(W_{i}\right)}_{j,:}^{\tau }||}_{2} \end{array}\right\}\\ +tr\left({\left(W^{\tau }\right)}^{T}\Omega^{\tau }W^{\tau }\right)+\lambda tr\left({\left(W^{\tau }\right)}^{T}U^{\tau }W^{\tau }\right). \end{array}$$

Eq. 35 can be further rewritten as:

(36) $$\begin{array}{c} \sum_{i=1}^{S}\{tr\left({\left(W_{i}^{\tau +1}\right)}^{T}C_{i}W_{i}^{\tau +1}\right)+\alpha g\left(F_{i}^{\tau +1}\right)+\vartheta_{i}^{q_{1}}\\ \sum_{j=1}^{n}||{\left(Z_{i}\right)}_{j,:}^{\tau +1}|{|}_{2}+\beta \sum_{j=1}^{n}||{\left(W_{i}\right)}_{j,:}^{\tau +1}|{|}_{2}\}\\ +tr\left({\left(W^{\tau +1}\right)}^{T}\Omega^{\tau +1}W^{\tau +1}\right)+\lambda tr\left(W^{\tau +1}{\left(W^{\tau +1}\right)}^{T}U^{\tau +1}\right)\\ -\frac{\lambda }{2}tr\left({\left(W^{\tau +1}{\left(W^{\tau +1}\right)}^{T}\right)}^{\frac{1}{2}}\right)+\frac{\lambda }{2}tr\left({\left(W^{\tau +1}{\left(W^{\tau +1}\right)}^{T}\right)}^{\frac{1}{2}}\right)\\ \leq \sum_{i=1}^{S}\{tr\left({\left(W_{i}^{\tau }\right)}^{T}C_{i}W_{i}^{l}\right)+\alpha g\left(F_{i}^{\tau }\right)+\vartheta_{i}^{q_{1}}\\ \sum_{j=1}^{n}||{\left(Z_{i}\right)}_{j,:}^{\tau }|{|}_{2}+\beta \sum_{j=1}^{n}||{\left(W_{i}\right)}_{j,:}^{\tau }|{|}_{2}\}\\ +tr\left({\left(W^{\tau }\right)}^{T}\Omega^{\tau }W^{\tau }\right)+\lambda tr\left(W^{\tau }{\left(W^{\tau }\right)}^{T}U^{\tau }\right)\\ -\frac{\lambda }{2}tr\left({\left(W^{\tau }{\left(W^{\tau }\right)}^{T}\right)}^{\frac{1}{2}}\right)+\frac{\lambda }{2}tr\left({\left(W^{\tau }{\left(W^{\tau }\right)}^{T}\right)}^{\frac{1}{2}}\right). \end{array}$$

Noting that $U^{l}=\frac{1}{2}{\left(W^{\tau }{\left(W^{\tau }\right)}^{T}\right)}^{-\frac{1}{2}}$ and according to Lemma 2, we get

(37) $$\begin{array}{c} \lambda tr\left(W^{\tau +1}{\left(W^{\tau +1}\right)}^{T}U^{\tau +1}\right)-\lambda tr\left({\left(W^{\tau +1}{\left(W^{\tau +1}\right)}^{T}\right)}^{\frac{1}{2}}\right)\\ \geq \lambda tr\left(W^{\tau }{\left(W^{\tau }\right)}^{T}U^{\tau }\right)-\lambda tr\left({\left(W^{\tau }{\left(W^{\tau }\right)}^{T}\right)}^{\frac{1}{2}}\right). \end{array}$$

Subtracting Eq. (37) from Eq. 36, we have

(38) $$\begin{array}{c} \sum_{i=1}^{S}\{tr\left({\left(W_{i}^{\tau +1}\right)}^{T}C_{i}W_{i}^{\tau +1}\right)+\alpha g\left(F_{i}^{\tau +1}\right)+\vartheta_{i}^{q_{1}}\\ \sum_{j=1}^{n}||{\left(Z_{i}\right)}_{j,:}^{\tau +1}|{|}_{2}+\beta \sum_{j=1}^{n}||{\left(W_{i}\right)}_{j,:}^{\tau +1}|{|}_{2}\}\\ +tr\left({\left(W^{\tau +1}\right)}^{T}\Omega^{\tau +1}W^{\tau +1}\right)+\lambda tr\left({\left(W^{\tau +1}{\left(W^{\tau +1}\right)}^{T}\right)}^{\frac{1}{2}}\right)\\ \leq \sum_{i=1}^{S}\{tr\left({\left(W_{i}^{\tau }\right)}^{T}C_{i}W_{i}^{\tau }\right)+\alpha g\left(F_{i}^{\tau }\right)+\vartheta_{i}^{q_{1}}\\ \sum_{j=1}^{n}||{\left(Z_{i}\right)}_{j,:}^{\tau }|{|}_{2}+\beta \sum_{j=1}^{n}||{\left(W_{i}\right)}_{j,:}^{\tau }|{|}_{2}\}\\ +tr\left({\left(W^{\tau }\right)}^{T}\Omega^{\tau }W^{\tau }\right)+\lambda tr\left({\left(W^{\tau }{\left(W^{\tau }\right)}^{T}\right)}^{\frac{1}{2}}\right). \end{array}$$

That is to say

(39) $$\begin{array}{c} \sum_{i=1}^{S}\{tr\left({\left(W_{i}^{\tau +1}\right)}^{T}C_{i}W_{i}^{\tau +1}\right)+\alpha g\left(F_{i}^{\tau +1}\right)+\vartheta_{i}^{q_{1}}\\ \left||K_{i}^{T}W_{i}^{\tau +1}-F_{i}^{\tau +1}|{|}_{2,1}+\beta ||W_{i}^{\tau +1}|{|}_{2,1}\right\}\\ +tr\left({\left(W^{\tau +1}\right)}^{T}\Omega^{\tau +1}W^{\tau +1}\right)+\lambda {||W^{\tau +1}||}_{*}\\ \leq \sum_{i=1}^{S}\{tr\left({\left(W_{i}^{\tau }\right)}^{T}C_{i}W_{i}^{\tau }\right)+\alpha g\left(F_{i}^{\tau }\right)+\vartheta_{i}^{q_{1}}\\ \left||K_{i}^{T}W_{i}^{\tau }-F_{i}^{\tau }|{|}_{2,1}+\beta ||W_{i}^{\tau }|{|}_{2,1}\right\}\\ +tr\left({\left(W^{\tau }\right)}^{T}\Omega^{\tau }W^{\tau }\right)+\lambda {||W^{\tau }||}_{*}. \end{array}$$

Hence, the theorem has been verified.

According to the optimization strategy of Algorithm 1, the objective function is monotonically decreasing in problem Eq. 10, so it is easiest to observe that the algorithm is convergent.

### Generalization

In this part, we derive an empirical bound for our method that shows how both MAF and PDS control the generalization performance under the situation of the squared loss *loss*(*a*, *b*) = (*a*−*b*)^2^. The main idea is to merge the domain scatter into the proven adaptive range for the distance difference (Ghifary et al., 2017).

Denote by *H*:={*h*: 𝒳 → 𝒴} a hypothesis class of functions in the RKHS ℋ, where 𝒳 is a compact set and 𝒴 is a label space. Given a loss function *loss*(⋅,⋅): 𝒴 × 𝒴 → R_+_ and a domain distribution 𝒟 over 𝒳, we denote by ℒ_𝒟_(*h*, h̊) = *E*_*x*∼𝒟_[*loss*(*h*(*x*), h̊(*x*))] the expected loss for the given two functions *h*, h̊ ∈ *H*. Then, the distance of domain difference between two distributions P and Q is defined as:

(40) $$disc\left(P,Q\right)=\underset{h,\text{h̊}\in H}{\text{sup}}\{{ℒ}_{P}\left(h,\text{h̊}\right)-{ℒ}_{Q}\left(h,\text{h̊}\right)\text{\}},$$

By the notation in Eq. 40, we can obtain domain generalization bounds by domain scatter. Let *f*_P_ and *f*_Q_ be the true labeling functions for domain P and Q, respectively, and $h_{P}^{*}:=\underset{h\in H}{\text{argmin}}{ℒ}_{P}\left(h,f_{P}\right)$ and $h_{Q}^{*}:=\underset{h\in H}{\text{argmin}}{ℒ}_{Q}\left(h,f_{Q}\right)$ be the minimizers. The following theorem provides adaptation bounds with PDS (….).

***Theorem 4 (adaptation bounds with PDS)*** (Ghifary et al., 2017): Denote by *H*:={*f* ∈ ℋ: 𝒳 → R, ||*f*||_ℋ_ ≤ 1 *and* ||*f*||_∞_ ≤ *r*} a class of functions in the RKHS ℋ and by $X_{𝒳}^{P}=\left(x_{1}^{s},\ldots ,x_{n_{s}}^{s}\right)∼P$ and $X_{𝒳}^{Q}=\left(x_{1}^{t},\ldots ,x_{n_{t}}^{t}\right)∼Q$ the source and target dataset, respectively. Let *loss*(⋅,⋅): 𝒴 × 𝒴 → [0, Υ] be a *q*−Lipschitz loss function, i.e., for all *a*, *b* ∈ 𝒴 × 𝒴, ||*loss*(*a*)−*loss*(*b*)|| = *q*||*a*−*b*||. Then, for any hypothesis *h* ∈ *H*, with probability of at least 1 − δ, the following generalization bound holds with the Rademacher complexity ${ℜ}_{X_{𝒳}^{P}}\left(H\right)$ over $X_{𝒳}^{P}$:

(41) $$\begin{array}{c} {ℒ}_{Q}\left(h,f_{Q}\right)-{ℒ}_{Q}\left(h_{Q}^{*},f_{Q}\right)\leq {ℒ}_{P}\left(h,h_{P}^{*}\right)+2q{ℜ}_{X_{𝒳}^{P}}\left(H\right)\\ +3\Upsilon \sqrt{\frac{\log\frac{2}{\delta }}{2n_{t}}}+8r\sqrt{\Xi_{\phi }\left(\left\{\mu_{Q},\mu_{P}\right\}\right)}+{ℒ}_{P}\left(h_{Q}^{*},h_{P}^{*}\right). \end{array}$$

Theorem 4 provides a generalization bound for DA by introducing PDS and Rademacher complexity that measures the level to which a class of functions can fit random noise. The Rademacher complexity measure is the basis of relating empirical loss with expected loss. From Theorem 4, for a successful DA, we shall make ${ℒ}_{P}\left(h_{P}^{*},h_{Q}^{*}\right)$ as small as possible. According to definition 3, the (squared) loss ${ℒ}_{P}\left(h_{P}^{*},h_{Q}^{*}\right)$ is essentially equivalent to MDD in some optimal RKHS. We then further provide the following adaptation bounds with PDS and MAF, which follows by Theorem 1 combined with Theorem 4.

***Theorem 5 (adaptation bounds with PDS and MAF):*** Denote by *H*:={*f* ∈ ℋ: 𝒳 → R, ||*f*||_ℋ_ ≤ 1 and ||*f*||_∞_ ≤ *r*} a class of functions in the RKHS ℋ and by $X_{𝒳}^{P}=\left(x_{1}^{s},\ldots ,x_{n_{s}}^{s}\right)∼P$ and $X_{𝒳}^{Q}=\left(x_{1}^{t},\ldots ,x_{n_{t}}^{t}\right)∼Q$ the source and target dataset, respectively. Let *loss*(⋅,⋅):𝒴 × 𝒴 →[0,Υ] be a *q*−Lipschitz loss function, i.e., for all *a*, *b* ∈ 𝒴 × 𝒴, ||*loss*(*a*)−*loss*(*b*)|| = *q*||*a*−*b*||. Denote by $W_{P}^{*},W_{Q}^{*}$ the optimal functions learnt from domain P and domain Q, respectively; then, for any hypothesis *h* ∈ *H*, with probability of at least 1−δ, the following generalization bound holds with the Rademacher complexity ${ℜ}_{X_{𝒳}^{P}}\left(H\right)$ over $X_{𝒳}^{P}$ :

(42) $$\begin{array}{c} {ℒ}_{Q}\left(h,f_{Q}\right)-{ℒ}_{Q}\left(h_{Q}^{*},f_{Q}\right)\leq {ℒ}_{P}\left(h,h_{P}^{*}\right)+2q{ℜ}_{X_{𝒳}^{P}}\left(H\right)\\ +3B\sqrt{\frac{\log\frac{2}{\delta }}{2n_{t}}}+8r\sqrt{\Xi_{\phi }\left(\left\{\mu_{Q},\mu_{P}\right\}\right)}+\Psi \left(\left\{W_{P}^{*},W_{Q}^{*}\right\}\right). \end{array}$$

Theorem 5 clearly shows that the projected domain scatter Ξ_ϕ_({μ_Q_, μ_P_}) and MAF $\Psi \left(\left\{W_{P}^{*},W_{Q}^{*}\right\}\right)$ can control the generalization performance of MACI with its empirical measure, that is, to minimize the PDS (or, alternatively, the distributional scatter discrepancy) and MAF (or model discrimination discrepancy) in our methods can effectively improve the generalization bound in the setting of MDA or domain generalization, which is also supported by the following real-world experiments.

## Experiments

In this section, to evaluate the effectiveness of MACI for emotion recognition, we compare it with several state-of-the-art methods on two benchmark datasets, i.e., DEAP (Koelstra et al., 2012) and SEED (Zheng and Lu, 2015), which are also widely adopted as benchmark datasets for EEG-based emotion recognition (Mansour et al., 2009). Since existing deep DA models have demonstrated to be very effective, mainly applied to the EEG-based emotion recognition problems (Lotfi and Akbarzadeh, 2014), we divide our experiments into two parts, i.e., comparisons with shallow (traditional) DA methods on those emotion recognition tasks mentioned above and comparisons with the deep (CNN-based) DA methods for EEG-based emotion recognition on several cross-datasets.

## Data Preparation

At present, there are some EEG datasets for emotional state research. In this article, we used the following two public datasets: DEAP (Koelstra et al., 2012) and SEED (Zheng and Lu, 2015). As reported in Zhong et al. (2020) and Lan et al. (2018), there is a significant difference between these two databases due to some technical aspects. We also adopted the same feature extraction strategy with that in Lan et al. (2018). More details about these two databases can be found in Lan et al. (2018).

In our experiments, differential entropy (DE) (Zhong et al., 2020) is employed as the feature of emotion recognition. In the literature (Shi et al., 2013; Zheng et al., 2015; Chai et al., 2016; Zheng and Lu, 2016; Chai et al., 2017; Lan et al., 2018; Zhong et al., 2020) about the DA emotion recognition based on EEG, DE features have been widely used. The details of DE are explained in Lan et al. (2018).

### Baseline Setting

We compare our MACI method with the following state-of-the-art (related) baselines for multi-source emotion recognition tasks. Besides this, we also report the emotion recognition results of MACI using several deep features:

• No adaptation baseline FSSL (Yang et al., 2013)
• Multi-kernel adaptation method: FastDAM (Duan et al., 2012c)^3^
• Multi-KT (Tommasi et al., 2014)^4^ : according to Tommasi et al. (2014), we here also use the *l*_2_−*norm* constraint on *p* in Multi-KT algorithm
• Adaptive SVM: A-SVM (Yang et al., 2007)^5^
• Domain selection machine (DSM) (Duan et al., 2012a)
• Deep DA methods: DAN (Long et al., 2015) and ReverseGrad (Ganin and Lempitsky, 2015).

For the baseline FSSL without adaptation and the multi-source adaptation method A-SVM, we just equally fuse the decision values of all base classifiers with each classifier learned on one source domain^6^.

In our MACI, only several vital parameters such as *q*_1_, *q*_2_, λ, α, and β in our model need to be predefined. Considering that parameter determination is a yet unaddressed open issue, we determine these parameters empirically as in our previous works (Tao et al., 2019). The parameters *q*_1_ and *q*_2_ play the same role in optimizing ϑ_*a*_ and η_*a*_ for preventing the trivial solution of these optimal variables. Since the larger *q*_1_ (or *q*_2_ ) would lead to the same weights with greater probability, we therefore empirically set *q*_1_ = *q*_2_ = 2 in our experiments in terms of the suggestion provided in Hou et al. (2017). Besides this, we discreetly choose the values of λ, α, and β by employing the grid search strategy in a heuristic way. Concretely, these regularization parameters are tuned from {10^−4^,10^−3^, …, 10^3^,10^4^}. Finally, we search and fine-tune the number of nearest neighbors *k* in the set {3,5,10,15,17} for constructing the affinity graph in MACI (also in FSSL). For our algorithm, the maximum iteration number is set as τ = 100.

For those nonlinear learning methods MACI, FastDAM, and Multi-KT, we borrow the Gaussian kernel [i.e., *K*_*i*,*j*_ = exp (−σ||*x*_*i*_−*x*_*j*_||^2^)] as the default kernel function, where σ is determined by setting it to be the reciprocal of feature dimension 1/*d*. Following the same practice in Duan et al. (2012a), we predefine each source weight $\gamma_{i}=\frac{\exp\left(-\delta Dist\left(X_{i}^{s},X\right)\right)}{\sum_{i}\exp\left(-\delta Dist\left(X_{i}^{s},X\right)\right)}$ ( *i* = 1, …, *S*) in FastDAM, where δ = 100.

#### Experiment I: Within-Dataset Emotion Recognition

It is worth noting that we may encounter difficulties with different subjects in EEG emotion recognition even if they belong to the same dataset because different subjects may have different EEG feature distributions due to personalized characteristics. Thereby, we may adopt the so-called leave-one-subject-out cross-validation strategy adopted also in Lan et al. (2018) to evaluate the performance of MACI on emotion recognition. Concretely, the left subject from the dataset of interest contributes to the target domain, and other subjects are constructed as the multi-source domains. We evaluate the multi-source adaptation performance of MACI compared with existing state-of-the-arts on SEED and DEAP, respectively.

There are totally 2,340 training data of 13 subjects with 60 data per class and 180 test data of each subject from three classes in DEAP. We extracted 2,775 samples consisting of 925 samples per class per class hour from each one of the 14 subjects in SEED, thus generating 38,850 training data from 14 subjects and 2,775 test samples from one target subject. Note that extant research (Chai et al., 2016, 2017; Zheng and Lu, 2016) have pointed out that it is almost impossible to train the DA methods by exploiting all training data from SEED due to the limitation of computational space. We thereby randomly sample 10% training data from SEED, i.e., 3,885 training data as the final multi-source domain data for all DA methods. We repeat each trial 10 times on SEED, and the final performance is the average of the results of 10 times.

#### Performance Comparison

We show in Table 2 the emotion recognition performance of MACI and several baselines on within-DEAP and within-SEED, respectively.

**TABLE 2:** Emotion recognition performance (mean % and SD %) of MACI and several baselines on within-datasets.

Method	DEAP		SEED							
			Session I		Session II		Session III		Average	
	Mean	SD	Mean	SD	Mean	SD	Mean	SD	Mean	SD
FSSL	40.17	4.36	57.96	6.85	48.79	5.47	57.45	9.09	53.78	6.96
Multi-KT	55.83	5.59	73.56	4.37	68.89	3.43	72.57	7.38	70.68	5.09
A-SVM	49.49	7.92	65.82	7.86	64.00	7.09	69.08	10.77	65.25	8.53
FastDAM	57.37	5.50	72.31	6.86	69.45	7.18	75.64	7.37	71.52	7.04
DSM	60.22	6.50	72.76	6.86	70.10	5.18	76.35	7.37	72.27	6.47
MACI	63.31	4.50	73.42	6.86	70.81	6.18	77.43	7.37	73.23	6.66
Upp Bnd of Chn Lvl	38.85		34.58		34.65		34.60		34.61	

As reported in Lan et al. (2018), the theoretical performance (or chance level) of random guessing is about 33.33%, which could be approached by real chance level when the number of training samples increase to infinity (Lan et al., 2018). As shown in Table 1, the baseline FSSL contributes 40.17% mean recognition accuracy on DEAP, which is very near to the random value. When there are finite samples, we obtain the empirical chance level by repeating the trials of the samples in question equipped with randomized class labels (Lan et al., 2018). The finally obtained chance levels with bound of 95% confidence interval are also recorded in Table 2. We can see from Table 2 that the accuracy of FSSL significantly exceeds the upper bound of the real chance level at 5% significance level. However, the relatively lower performance of FSSL still indicates that emotion recognition with DA technique is imperative when there exists substantial divergence between the feature distributions of different subjects.

Almost all DA methods yield better recognition performance than FSSL for DEAP. Our MACI achieves the best performance (about 23.14% gains in performance over FSSL), closely followed by DSM. Note that though we acquired the relatively significant improvement measured by *t*-test with *p*-value > 0.05, the total recognition accuracy is still inferior. On SEED, FSSL achieves 53.78% average recognition accuracy over three sessions, which obviously surpasses the upper bound of the chance level. Several multi-source adaptation methods, i.e., Multi-KT, FastDAM, and DSM, undoubtedly obtain more performance gains than FSSL on SEED. The proposed method MACI still effectively boost the mean recognition accuracy up to 73.23% under *t*-test with *p*-value > 0.05, which still demonstrates the best performance on SEED. It is worthy to note that all methods including FSSL work more effectively on SEED than on DEAP. This interesting observation is partially consistent with that in Lan et al. (2018). A possible explanation may be that the so-called negative transfer prevented the effective application of DA techniques in DEAP since larger discrepancies among different subjects may exist in DEAP than in SEED (Mansour et al., 2009; Lan et al., 2018).

#### Number of Source Samples

**FIGURE 1:**
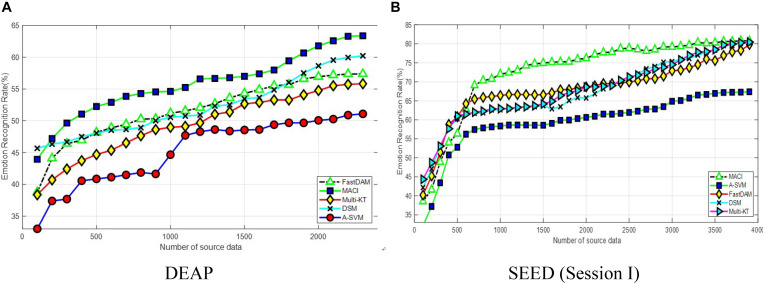
Classification accuracy with varying numbers of source samples on **(A)** DEAP and **(B)** SEED (session I).

Figure 1 presents the effect of varying the number of source samples. The source dataset size varies from 100 to 2,300 on DEAP and 100 to 3,800 on SEED, respectively. It can be seen from the curves in Figure 1 that all methods manifest the same trend of upgrade in the figure. This shows that larger source data is beneficial to improve the learning performance. It is worthy to note that the performance of MACI can be smoothly and steadily improved with the increase of the source samples, while other DA methods may only achieve satisfactory performance when the source samples are relatively large, i.e., larger than 500. In addition, A-SVM obtains the least performance on two datasets due to the so-called negative transfer issue in DA.

On DEAP, MACI, and DSM maintain a better accuracy than other methods with less than 500 source domain samples. DSM outperforms Multi-KT and FastDAM when the number of target training samples is relatively large due to properly choosing the weights to assign to each subject. Our method MACI obtains even more gains over DSM when the number of source samples is increasing asymptotically. The reason may be that, except for the use of correlation information among subjects, MACI can effectively select the most related sources with the optimally weighted multi-source adaptation regularization. Besides that, partial experimental results also show that FastDAM could occasion the “negative transfer” issue with the MMD-based weights assigned to all sources, which would deteriorate its performance. On SEED, the accuracy flattens at above 3,000 source samples. When the number of source samples increases to 3,500, MACI, FastDAM, and Multi-KT asymptotically approach a similar performance. From this point onwards, MACI, FastDAM, and Multi-KT perform similarly if we have sufficient source data.

#### Multi-Kernel Learning

**FIGURE 2:**
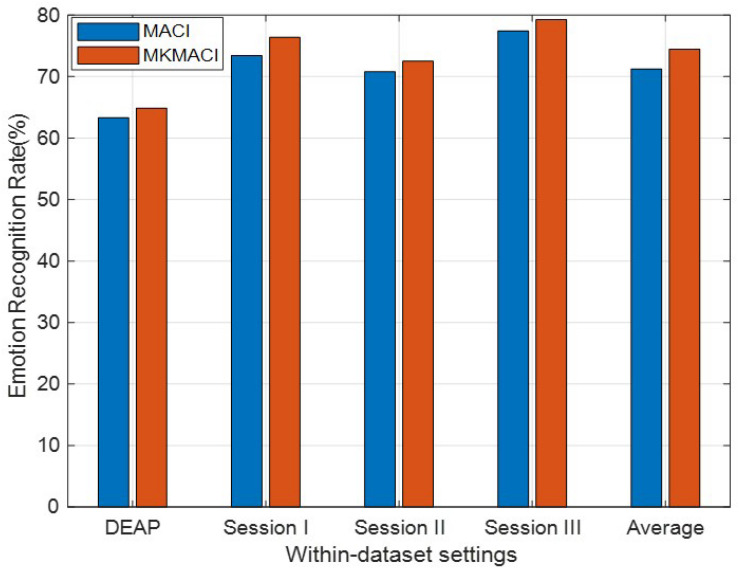
Domain adaptation emotion recognition on within-dataset with multi-kernel learning.

We further evaluate the effectiveness of our method with different kernel functions (called MKMACI for short) for each source domain. Given the empirical kernel mapping set ${\left\{\phi_{a}\right\}}_{a=1}^{℧}$, each mapping *X*_*a*_ into a different kernel space, we can integrate them orthogonally to the final space by concatenation, i.e., $\tilde{\phi }\left(x_{i}\right)={\left[\phi_{1}{\left(x_{i}\right)}^{T},\phi_{2}{\left(x_{i}\right)}^{T},\ldots ,\phi_{℧}{\left(x_{i}\right)}^{T}\right]}^{T}\in R^{℧n_{a}}$, for *x*_*i*_ ∈ *X*_*a*_. The final kernel matrix in this new space is defined as *K*_*new*_ = [K̊_1_; K̊_2_; …; K̊_℧_], where K̊_*i*_ is the kernel matrix in the *i*−th feature space. Therefore, besides the above-mentioned Gaussian kernel, we additionally employ another three types of kernels in MKMACI: Laplacian kernel $K_{ij}=\exp\left(-\sqrt{\sigma }||x_{i}-x_{j}||\right)$, inverse square distance kernel *K*_*ij*_ = 1/(1 + σ||*x*_*i*_−*x*_*j*_||^2^), and inverse distance kernel $K_{ij}=1/\left(1+\sqrt{\sigma }||x_{i}-x_{j}||\right)$. It can be clearly seen in Figure 2 that MKMACI is obviously better than MACI in terms of mean accuracies in all cases, which justifies that the multi-kernel trick can improve the quality of DA emotion recognition on within-datasets.

### Experiment II: Cross-Dataset Emotion Recognition

Note that cross-dataset emotion recognition is more challenging in terms of the differences in acquisition and participant characteristics and behaviors. In the preceding experiments, we demonstrate the performance comparison of our method with other DA methods with the within-dataset (i.e., cross-subject) setting. We will, in this part, further evaluate the consistent effectiveness of MACI when performed on cross-dataset adaptation. In this scenario of experiment, we constructed multiple different schemes by sampling the training dataset and test dataset, respectively, with different EEG instruments and emotional stimuli. We therefore set up six trial settings, i.e., *DEAP* → *SEED I*, *DEAP* → *SEED II*, *DEAP* → *SEED III*, *SEED I* → *DEAP*, *SEED II* → *DEAP*, and *SEED III* → *DEAP*, to justify the effectiveness of MAC on cross-dataset emotion recognition. In the context, we denote A → B by adaptation from dataset A to dataset B. For simplicity of expression, we respectively, coined SEED I, SEED II, and SEED III as the dataset of session I, session II, and session III in the database SEED.

In universe DA, a commonly used hypothesis is that the feature space of both source and target domains is the same. Consequently, only 32 channels between SEED and DEAP are employed to formulate a 160 dimensional feature space for both training and test datasets. In the first three experimental settings, there are 180 × 14 = 2,520 source samples from DEAP and 2,775 target samples from three different sessions in SEED. We evaluate the recognition accuracy for each subject in each session and report the final experimental results based on the mean over 15 subjects from SEED. In the other experimental settings, a total of 2,775 × 15 = 41,625 source samples from SEED are regarded as training datasets, and 180 samples contributed from DEAP are test dataset. We then evaluate the recognition accuracy of individual subjects in DEAP, and the results are recorded with the average over 14 subjects. We randomly sample 10% of the source data (4,162 samples) as the actual training data due to the limitation of memory (Shi et al., 2013; Zheng et al., 2015; Chai et al., 2016, 2017; Zheng and Lu, 2016; Lan et al., 2018; Zhong et al., 2020). Under each setting, we conduct the trial repeatedly 10 times and record the average performance over these 10 times.

#### Performance Comparison

We record the mean experimental results on six cross-dataset settings in Table 3, from which we can observe that the performance of the baseline FSSL is inferior to the upper bound of chance level with 95% confidence interval, that is, the baseline performance is almost close to the random guess with 5% significance level. This indicates that there exist larger distribution divergences between two datasets as well as the variance among different subjects than that in within-dataset. The importance of DA would be indispensable in this scenario. This is justified by the observation in Table 3 that all DA methods outperform the baseline FSSL since DA could potentially reduce the technical discrepancies in cross-dataset applications. In most cases, MACI is found to be the best-performing method in the cross-dataset DA settings. In some scenario, Multi-KT and FastDAM occasionally obtain the best performance. A noticeable phenomenon can be observed in Table 3, such that the mean recognition accuracies of all methods are correspondingly worse than that in Table 2 obtained on within-dataset due to the larger distribution discrepancy between different datasets.

**TABLE 3:** The recognition accuracy (mean%) with cross-dataset settings.

Method	DEAP→SEED I	DEAP→SEED II	DEAP→SEED III	SEED I→DEAP	SEED II→DEAP	SEED III→DEAP
FSSL	32.42	33.71	34.47	33.57	32.99	32.51
A-SVM	55.86	58.48	60.84	39.68	40.08	39.53
FastDAM	65.72	62.68	66.21	48.40	49.90	47.46
DSM	68.47	64.68	64.33	50.22	51.44	50.46
Multi-KT	67.74	65.51	64.65	48.73	52.16	51.27
MACI	69.36	67.60	65.43	54.37	51.88	51.76
Upp Bnd of Chn Lvl.	34.68	34.72	34.74	38.35	38.38	38.44

#### Multi-Source Adaptation

**FIGURE 3:**
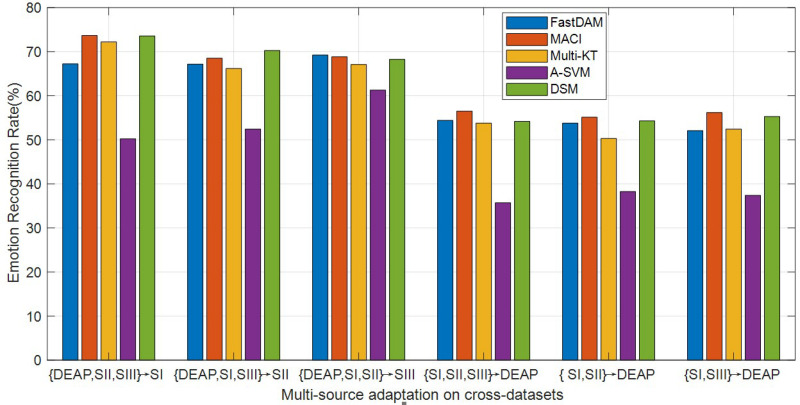
Multi-source adaptation emotion recognition accuracy (SI, session I; SII, session II; SIII, session III).

In practical DA applications, one may expect that the number of prior sources grow in time, which would incur the so-called scalability issue. In this problem, it is necessary to explore the reliability of each prior source for the specific task (Tao et al., 2019). To this end, we additionally conduct multi-source adaptation trials on several cross-dataset settings. The average results of MACI, DSM, Multi-KT, FastDAM, and A-SVM with the average prior model are reported in Figure 3.

It can be seen from the curves in Figure 3 that A-SVM is still worse than the other DA methods in most cases in that it is difficult for A-SVM to minimize the between-domain distribution distance when the distribution varies greatly between domains. The accuracies of A-SVM tend to be downgraded when the number of sources is increasing in some cases, suggesting that negative transfer may have happened in A-SVM. MACI obtains a relatively much better performance in most cases, which demonstrates that our algorithm can improve the emotion recognition performance on cross-dataset. All methods except A-SVM manifest the same trend of upgrade with the increase of sources, and the accuracy improvements are significant with respect to that of within-dataset settings. This shows that utilizing the limited sources is beneficial to improve the learning performance. In addition, MACI and DSM usually outperform other DA methods due to properly choosing the weights to assign to each source. Our method MACI obtains even more gains over DSM, which may be attributed to the utilization of correlation information among sources in that MACI can effectively select the most related source domains with the optimally weighted multi-source adaptation regularization.

#### Adaptation With Deep Features

In the past decade, deep learning attracts more and more attention due to its powerful representation ability and dramatic improvement over the traditional shallow methods. We therefore additionally compare our MACI method with the recently proposed deep transfer learning models DAN and ReverseGrad for cross-dataset emotion recognition using deeply extracted features in multi-source adaptation settings.

In our MACI, we can tackle the problem of deep DA with two steps: firstly, a higher-level feature extraction is learnt in an unsupervised fashion from all available domains using the popular deep architectures [e.g., VGG16 (Simonyan and Zisserman, 2014) or DAN]; secondly, our MACI is trained on the transformed data of all domains and then used to test the target domain. For fair comparison, however, we follow the experimental setup in Zhou et al. (2018) and Zhu et al. (2017). Specifically, we first fine-tune pretrained deep models (e.g., VGG16, DAN, and ReverseGrad) by using the labeled samples in the source domain and then use these fine-tuned CNN models to extract the features from EEG in both source and target domains. Finally, we perform emotion recognition using MACI on these deeply extracted features. In the context of our experiments, we denote our methods with different deep models as MACI + VGG16, MACI + DAN, and MACI + ReverseGrad, respectively. As for DAN and ReverseGrad, we use their released source codes and fine-tune the pre-trained deep models by using the suggested parameters in Long et al. (2015) and Ganin and Lempitsky (2015), respectively.

**FIGURE 4:**
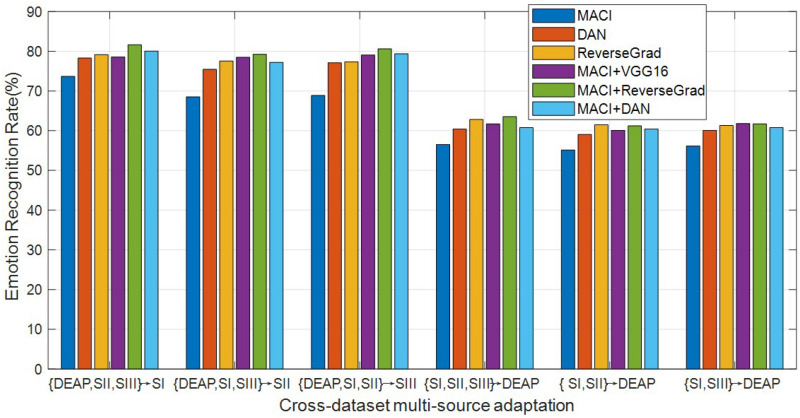
Emotion recognition accuracies (%) of different methods using deeply extracted features (SI, session I; SII: session II; SIII, session III).

All experimental results are reported in Figure 4. As can be seen from this plot, the deep transfer learning methods are originally proposed to learn domain-invariant features, while our proposed method aims to improve the cross-domain generalization ability, namely, their methods focus on feature learning, while our work focuses on classification, so our proposed method can be used to further improve the recognition accuracies by co-learning the source classifiers with the features extracted by deep models, i.e., VGG16, DAN, and ReveseGrad. This indicates that the classification-level constraint can preserve all source discriminative structures for the guidance of target data classification, which demonstrates the effectiveness of MACI framework. From the plot bars of Figure 4, it can be observed that MACI + DAN consistently outperforms DAN, while MACI + ReverseGrad is consistently better than ReveseGrad, which demonstrates that our MACI method is complementary to the two deep transfer learning methods DAN and ReveseGrad by exploiting the correlation statistics to further enhance the generalization ability across domains.

#### Parameter Impact on MACI

There are mainly three model parameters to be tuned in our method, i.e., λ, β, and α. Note that larger α would make the predicted label matrix better meet the expected needs, thus with better results being achieved. Consequently, we empirically set α = 10^3^ in the following experiments. We firstly explore to set the extreme values of different parameters for validating the importance of each component in our framework. Specifically, we denote MACI without the feature selection (i.e., β = 0) by MACI_NF and MACI with λ = η_*a*_ = 0 by MACI_NS, which ignores correlation information among multiple sources. These settings are evaluated on cross-dataset settings for multi-source adaptation tasks. From Table 4, we can observe that MACI can be significantly improved from MACI_NS by exploiting the correlation information among multiple sources. Besides this, the performance of MACI_NF is slightly weaker than MACI, that is, MACI would degrade when the feature selection function is omitted. A possible reason may be that the features of EEG represented by DE introduced some noise/outlier data. In this case, the feature selection in MACI possesses indispensable importance for robust DA learning. In sum, the utilization of correlation knowledge among sources and features could make MACI further boost its performance in cross-dataset emotion recognition applications. It is this argument that constitutes the basic principle of our MACI framework.

**TABLE 4:** Cross-dataset emotion recognition rates with different strategies of parameter settings.

Method	{DEAP, SII, SIII}→SI	{DEAP, SI, SIII}→SII	{DEAP, SI, SII}→SIII	{SI, SII, SIII}→DEAP	{SI, SII}→DEAP	{SI, SIII}→DEAP
MACI_NF	73.32	67.24	69.78	54.62	55.04	54.39
MACI_NS	69.88	65.31	66.07	52.92	53.44	52.81
MACI	73.69	68.52	68.85	56.52	55.12	56.17

*SI, session I; SII, session II; SIII, session III.*

## Conclusion

In this work, we explore to cope with the cross-dataset emotion recognition where existing BCI methods cannot work well. To this end, we proposed an effective multi-source co-adaptation framework (MACI) for EEG-based emotion recognition mainly by leveraging correlation knowledge among sources and features in the objective function, which dampens unimportant evidence (within features and between sources) and amplifies useful knowledge. In MACI, multiple domain-invariant classification functions corresponding to different sources are co-learned by bridging both statistical and semantic distribution discrepancy between source and target domains, thus making MACI utilize the correlated knowledge among multiple sources by exploiting the developed correlation metric function. A large number of experimental results conducted on two publicly available EEG datasets show that MACI are much better than several representative baseline methods and provide the state-of-the-art performance on within/cross-dataset emotion recognition in most cases. This demonstrates the effectiveness of MACI in addressing feature distribution discrepancy between individual subjects as well as different datasets due to technical discrepancies.

To boost the efficiency of our method, however, a more efficient iterative algorithm would be developed or further elaborated in our future works. Besides this, the pseudo-labels strategy (i.e., iteratively updating target label matrix in the training stage) for bridging semantic distribution discrepancy between different domains would be unreliable or even misleading in training. This therefore arouses another challenge, i.e., how to effectively infer and incorporate target labels in unsupervised DA, which would be an urgent and valuable work in our future research.

## Data Availability Statement

Publicly available datasets were analyzed in this study. This data can be found here: http://epileptologie-bonn.de/cms/upload/workgroup/lehnertz/eegdata.html.

## Ethics Statement

Ethical review and approval was not required for the study on human participants in accordance with the local legislation and institutional requirements. Written informed consent for participation was not required for this study in accordance with the national legislation and the institutional requirements.

## Author Contributions

Both authors listed have made a substantial, direct and intellectual contribution to the work, and approved it for publication.

## Conflict of Interest

The authors declare that the research was conducted in the absence of any commercial or financial relationships that could be construed as a potential conflict of interest.

## Footnotes

1 It is important to note that the feature mapping function ϕ_*a*_ (_1≤a≤S_) with respect to each source domain can be completely different from each other.

2 For maximal consistency between *F^T^F* and *I*_*c*_, the parameter ζ should be set as a relatively large value. In our experiments, we therefore empirically set ζ = 10^3^ without loss of performance to some extent.

3 The code is available from http://vc.sce.ntu.edu.sg/transfer_learning_domain_adaptation_data/DAM-TNNLS2012.html.

4 We use the code available from http://homes.esat.kuleuven.be/~ttommasi/source_code_CVPR10.html.

5 The MATLAB code is available online http://www.robots.ox.ac.uk/~{}vgg/software/tabularasa/.

6 For each source domain, we train one SVM by using the corresponding labeled samples. Then, for each test instance *x*, the decision values from *p* SVM classifiers are converted into the probability values by using the sigmoid function [i.e., g(*t*) = 1/(1 + exp(−*t*)]. Finally, we average the *p* probability values as the final prediction of the test instance *x*.
